# Insights into the Electronic and Structural Properties of Cellulose and Amylose: A Comparative Force Field Study

**DOI:** 10.1021/acs.jpcb.5c07277

**Published:** 2026-01-06

**Authors:** Esmat Mohammadi, Justin A. Lemkul

**Affiliations:** † Department of Chemical Engineering, 1757 Virginia Tech, Blacksburg, Virginia 24061, United States; ‡ Department of Biochemistry, Virginia Tech, Blacksburg, Virginia 24061, United States; § Center for Drug Discovery, Virginia Tech, Blacksburg, Virginia 24061, United States

## Abstract

Amylose and cellulose are important biopolymers with diverse applications in biotechnology and materials science. Understanding their structural, dynamic, and solvation properties at the molecular level is critical for harnessing their potential. This study investigates the electronic and structural properties of single-chain cellulose and single- and double-chain amylose in aqueous solution using molecular dynamics simulations with both nonpolarizable (CHARMM) and polarizable (Drude) force fields. CHARMM simulations show stable hydrogen bonding between amylose and water, higher glucose ring dipole moments, increased rigidity, adoption of chair conformations, and less variation in dihedral angles. In contrast, Drude simulations captured dynamic electronic polarization, enhanced conformational flexibility, and resulted in heterogeneous inter- and intramolecular hydrogen bonds. For cellulose, structural and solvation behaviors were largely similar between CHARMM and Drude. These findings highlight molecular interactions and solvation dynamics of amylose and cellulose, with potential relevance in materials science and biotechnology.

## Graphical abstract

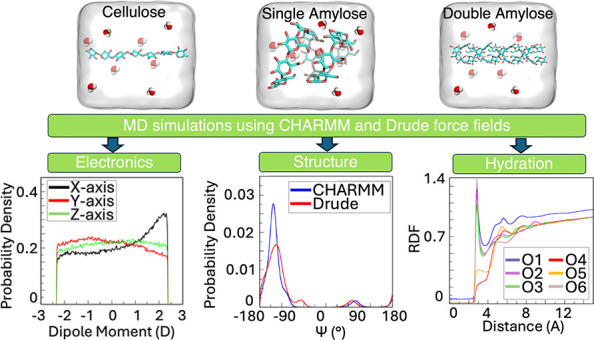

## Introduction

Carbohydrates comprise structurally diverse molecules with relevance to chemistry, biology, materials science, and related fields.^1,2^ In biological studies, carbohydrates, along with nucleic acids and proteins, play critical functional roles and influence numerous physiological processes.^3^ Consequently, the study of carbohydrates has garnered considerable attention due to their relevance to fundamental biochemical mechanisms and potential developments in the pharmaceutical field.^3,4^ Two prototypical polysaccharides, amylose and cellulose, serve as a key example of the need to study carbohydrates due to their different structures and functions. Amylose, with its helical shape and role in plant energy storage, consists of linear glucose units linked by α-1,4 glycosidic linkages.^5–7^ Cellulose, which provides structural support in plant cell walls, consists of linear glucose chains linked by β-1,4 glycosidic linkages that form crystalline fibrils.^8,9^ These polysaccharides differ only in the stereochemistry of their glycosidic linkages, which confers these different properties. Beyond structural differences, amylose is digestible by human enzymes, while cellulose is not due to its β-linkages.^10,11^

The behavior of carbohydrates and polysaccharides in water is of specific significance because the dynamic structure and properties of carbohydrates are strongly dependent on their interactions with water.^12–14^ These interactions play a crucial role in many biological processes, such as enzyme recognition and molecular transport.^15,16^ Furthermore, for industrial use, knowledge of carbohydrate behavior in water is essential for the design of biocompatible materials, such as hydrogels for drug delivery and wound healing.^17,18^ Overall, elucidation of the intricate interactions of carbohydrates and polysaccharides with water highlights the role of carbohydrates in biological systems and enables the development of new materials with diverse applications.

Molecular dynamics (MD) simulations have long been an important tool in exploring the behavior and interactions of biomolecules, including carbohydrates, at the atomic level.^19,20^ In carbohydrate science, MD simulations have been particularly valuable for understanding carbohydrate dynamics in water, probing their conformational flexibility, and analyzing their interactions with other biomolecules, including proteins and nucleic acids.^21^ These insights are invaluable to applications ranging from drug design to biomaterials engineering.^22,23^

The accuracy and reliability of MD simulations depend strongly on the quality of force field (FF) parameters, which define the interactions between atoms and molecules within the system being simulated.^24,25^ Specifically, FFs define the potential energy surface responsible for driving the molecular motion and interactions being simulated in MD simulations.^26^ Nonpolarizable FFs such as CHARMM,^27^ GROMOS,^28,29^ OPLS-AA,^30^ and GLYCAM06 ^31^ are commonly employed in molecular simulations of carbohydrates and polysaccharides. However, their reliance on fixed partial charges limits their ability to accurately model the intricate environments found in biological systems. For example, quantum mechanical (QM) studies^32,33^ have demonstrated that MD simulations utilizing nonpolarizable FFs struggle to adequately balance both inter- and intramolecular phenomena such as solvent effects, counterion interactions, hydrogen bonding, and molecular dipole variations, which involve complex charge redistributions. In response, the adoption of polarizable FFs capable of incorporating polarization serves to bridge the gap between QM investigations and classical MD simulations.^34^

The Drude polarizable FF, based on the classical Drude oscillator model, introduces additional auxiliary particles with negative charge to model electronic degrees of freedom and account for polarization effects, enabling explicit modeling of induced dipoles.^35–37^ This convention proves particularly useful in simulating complex systems with strong electrostatic interactions. However, the parametrization of the Drude FF is challenging, requiring care to avoid overpolarization and extensive testing because of its increased complexity.^36^ Parametrizing carbohydrates is particularly challenging due to dynamic ring puckering, the interdependence of neighboring hydroxyl groups, and the sensitivity of glycosidic linkages to electronic effects. Significant efforts have gone into developing the current Drude polarizable FF for carbohydrates. For instance, laying a foundation for carbohydrate modeling using polarizable FFs, Drude parameters for hexopyranoses were derived and validated against quantum mechanical data. These parameters were tested against dipole moments, vibrational spectra, and torsional profiles to accurately reproduce conformational energetics and molecular polarizability.^38^ Additional work expanded the Drude polarizable FF to enable modeling of polysaccharides containing other monosaccharides such as pyranose and furanose. Parametrization of these monosaccharides was based on QM potential energy scans, dipole moments, and molecular polarizabilities, resulting in a model that agrees well with both QM and experimental data, and improves upon the additive CHARMM36 carbohydrate FF.^39^ Further development extended the parameters to include both *N*- and *O*-linked glycoproteins, where the dihedral terms were optimized using solution NMR *J*-coupling data. This model was validated against several glycopeptides and glycoproteins, enabling compatibility between carbohydrate and protein components in polarizable MD simulations.^40^ Altogether, these developments, along with other studies on the Drude FF development for carbohydrates,^34,41^ have significantly advanced the ability to simulate carbohydrate systems with greater accuracy, particularly in capturing the delicate balance of electronic effects that govern their structural and dynamic properties.

Cellulose and amylose, which are key biopolymers with wide applications in material science and biotechnology, must be understood at the molecular level to harness their potential. Computational studies have been instrumental in elucidating their structural, conformational, and thermodynamic properties. For amylose, MD simulations have revealed its flexibility and its ability to adopt a helical conformation when complexed with fatty acids, such as linoleic acid, providing insights into its solubility and interactions in aqueous environments.^42^ Other studies have examined the flexibility of single-chain amylose in different solvents, revealing solvent-dependent helical stability and chain extension, which are critical for understanding its behavior in processing and biological environments.^14,43,44^ In the case of cellulose, atomistic simulations have characterized both its crystalline and amorphous phases, highlighting differences in chain conformations, torsional flexibility, and ring puckering.^45^ The crystalline phase of cellulose exhibits well-defined hydrogen bonding networks and structural parameters consistent with experimental data, while amorphous regions display greater conformational variability and distinct hydrogen bonding patterns that influence mechanical and solvation properties.^45^ Additionally, MD simulations have explored the twisting behavior of cellulose microfibrils, revealing that this phenomenon is driven by van der Waals interactions and modulated by intrachain hydrogen bonding and solvent effects.^46^ Other investigations have modeled cellulose hydration and swelling, demonstrating that water content affects both chain mobility and the stability of hydrogen bond networks.^47^ For instance, studies have shown that water molecules can disrupt the hydrogen bonding between cellulose chains, leading to increased molecular mobility and swelling.^47^ These additive FF studies have advanced our understanding of amylose and cellulose but may be limited by their fixed-charge treatment of electrostatics, which overlooks polarization effects that may be important for modeling the delicate balance between intramolecular hydrogen bonding and interactions with water. The Drude polarizable FF overcomes this limitation by explicitly modeling induced dipoles, offering a more accurate depiction of solvation, hydrogen bonding, and molecular interactions. Thus, it is expected that such a convention will lead to better predictions of structure and dynamics of these carbohydrates.

Here, we have investigated the behavior of amylose and cellulose in water through MD simulations with nonpolarizable (CHARMM36) and polarizable (Drude) FFs. Solvation has been studied through the analysis of parameters like radial distribution function and hydrogen bonding patterns. Comparing results from both FFs allowed us to highlight the influence of electrostatic interactions on solvation and helical structure. Structural analysis, e.g., radius of gyration and dihedral angle analysis, provided descriptions of amylose and cellulose conformations with atomistic resolution. The use of the Drude polarizable FF allowed for a quantitative description of electric fields around cellulose and amylose, revealing details of their electrostatic interactions with solvating water molecules. This approach offers a combined picture of the dynamic behavior and solvation properties of these biopolymers.

## Methods and Computational Details

### Systems Studied

In this study, we employed both CHARMM36 and Drude FFs to explore the behavior of single-stranded and double-stranded amylose [α(1→4)-d-glucopyranan] and a single-stranded cellulose [β(1→4)-d-glucopyranan] chain. Both polysaccharides contained 12 units of α-d-glucopyranose or β-d-glucopyranose, respectively. All the systems of amylose and cellulose chains were solvated in a box of water. The choice of 12-unit oligomers is consistent with previous MD studies of amylose and cellulose, which have employed chain lengths ranging from 9 to 30 sugar units to probe intrinsic conformational tendencies in dilute aqueous solution.^44,48,49^

To generate the initial geometry of a single amylose chain, we used the CHARMM internal coordinate builder and dihedral values of QM-optimized disaccharides. Additionally, we focused on investigating ideal amylose helices, known to form upon interaction with various molecules such as fatty acids.^44^ To this end, we investigated two distinct amylose structures with restrained dihedral angles (Φ, O5_*i*_-C1_*i*_-O4_*i–1*_-C4_*i–1*_, and Ψ, C1_*i*_-O4_*i–1*_-C4_*i–1*_-C5_*i–1*_). Our goal was to create amylose chains with channel-like central cavities capable of accommodating other molecules. This approach allowed us to compare the properties of water molecules both confined within the helical structure and in the surrounding environment. It also provided insights into the electronic properties, such as the dipole moment and electric field, induced by the amylose chain in its ideal helical conformation when interacting with other molecules. We constructed these initial coordinates using the CHARMM internal coordinate builder with manually specified dihedral values for Φ and Ψ. For the first restrained amylose system, the dihedral angles were taken from an experimental crystal structure of cycloamylose, with values shown in Table 1.^50^ For the second restrained amylose system, we extracted a single amylose chain from an experimental crystal structure of a double-helix amylose system.^51^ The Φ and Ψ dihedral angles were restrained at the experimental values throughout the simulation.^51^

**Table 1:** Simulations Descriptions for Amylose and Cellulose Systems

Label	Simulated chain	Number of water molecules	Simulation box size (Å)	Simulation time (ns)
**Amylose unrestrained**	Single-stranded amylose without any restraints	10723	68	1000
**Amylose restrained 1**	Single-stranded amylose with restrained dihedrals (Φ = 103° and Ψ = 115°)	1924	39	200
**Amylose restrained 2**	Single-stranded amylose extracted from double helix system with restrained dihedrals (Φ = 85,92° and Ψ = −145°, –153°)	8965	64	200
**Amylose double helix**	Double-stranded amylose	8886	64	1000
**Cellulose**	Single-stranded cellulose	18153	81	1000

Furthermore, we performed simulations on a double-helix crystal structure composed of two amylose chains.^51^ This structural arrangement was derived from an experimental study detailing the three-dimensional configuration of the crystalline segment of A-starch.^51^ Each chain consisted of 12 glucopyranose residues distributed across two left-handed, parallel-stranded double helices. The details of the MD simulations conducted for the amylose and cellulose systems are outlined in Table 1. Renderings of the initial structures of the amylose and cellulose systems are shown in Figure 1.

**Figure 1:**
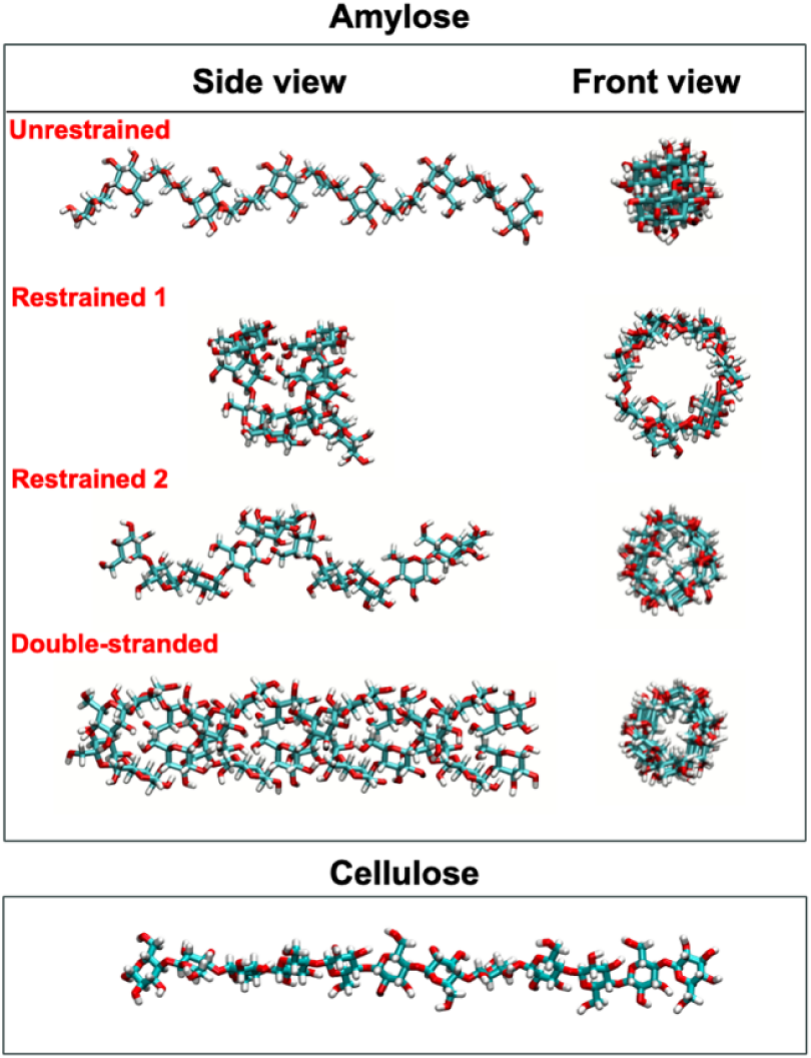
Snapshots of the side and front views of different amylose and cellulose systems studied.

### General MD Protocol

The initial setup applied the CHARMM36 FF to generate the coordinates and prepare the systems.^27^ Each amylose or cellulose chain was centered in a cubic box of TIP3P water molecules.^52–54^ Energy minimization was carried out using CHARMM36 FF,^55^ utilizing 500 steps of steepest descent minimization followed by 500 steps of adopted-basis Newton–Raphson minimization. Following minimization, a 1 ns equilibration was conducted under an NPT ensemble using OpenMM 7.7.0.^56^ During this period, position restraints were applied to all non-hydrogen atoms with a force constant of 500 kJ mol^–1^ nm^2^. Temperature was maintained at 298 K using the Langevin thermostat method and pressure set to 1 atm employing the Monte Carlo barostat method.^57,58^ Periodic boundary conditions were imposed in all dimensions, and the short-range van der Waals forces were smoothly switched to zero over a distance of 10–12 Å. Electrostatic interactions were computed using the particle mesh Ewald (PME) method with a real-space cutoff of 12 Å.^59^ Bonds to hydrogen atoms were constrained using SHAKE algorithm,^60^ allowing for a time step of 2 fs in CHARMM simulations.

After the CHARMM equilibration phase, the systems were transformed to the Drude polarizable model. This process involved adding Drude oscillators and lone pairs into the equilibrated coordinates. Simultaneously, the TIP3P water molecules were replaced with the polarizable SWM4-HLJ model.^61^ The relaxation of Drude oscillators was achieved through 1000 steps of steepest descent minimization, followed by 500 steps of adopted-basis Newton–Raphson energy minimization. The Drude systems were then equilibrated under an NPT ensemble, with temperature and pressure maintained at 298 K and 1 atm, respectively, using the same algorithms noted above for the CHARMM systems. The short-range Lennard-Jones potential was smoothly switched to zero from 10–12 Å, and electrostatic forces were calculated using PME.^59^ The polarizable systems were subjected to the same harmonic position restraints and bond constraints as the additive systems, although the integration time step was set to 1 fs to account for the high-frequency oscillations of Drude-atom bonds. A 1 ns equilibration period was employed for the polarizable systems.

Upon completion of equilibration, the production simulations were performed using OpenMM, maintaining the NPT ensemble with the previously described thermostat and barostat settings. The durations of the production simulations are provided in Table 1. For each system, three independent replicate simulations were produced by generating different, random velocities at the outset of equilibration.

### Clustering Analysis

To investigate the sampled conformational diversity across the simulations, we performed clustering analysis employing the MDANCE toolkit, which is used to analyze MD trajectories using stable, ensemble-based methods for clustering.^62^ For this work, we applied *k*-means *N*-Ary Natural Initiation (NANI) algorithm to identify dominant structural states. NANI is a centroid initialization method that enhances standard *k*-means clustering by emphasizing high-density regions of conformational space and selecting diverse initial cluster centers. Contrary to traditional stochastic approaches, NANI is deterministic and provides reproducible results when used with the same data, enhancing clustering accuracy and convergence efficiency.

To determine the appropriate number of clusters, we used the Davies-Bouldin (DB) index, which is a metric for evaluating clustering algorithms. To choose the appropriate number of clusters, two criteria are supported in MDANCE: (1) the lowest DB value and (2) the point corresponding to the maximum second derivative of the DB curve. In our case, we chose the number of clusters based on the lowest DB value, which helps to capture the most clearly defined conformational groupings. Prior to clustering, each of the trajectories was also aligned to a reference frame to remove global translational and rotational motion. Structural similarity was quantified in terms of root-mean-square deviation (RMSD) of heavy atoms, and the representative conformations were defined as the structures nearest the centroid of each cluster in RMSD space.

## Results and Discussion

The principal aims of our work were to characterize amylose and cellulose behavior at the molecular level and to compare the properties produced by the CHARMM and Drude FFs to quantify the inclusion of electronic polarization on dynamics and molecular interactions. Our approach explored single-chained cellulose and different conformations of amylose to systematically analyze the structural, hydration, and electronic characteristics within and surrounding these polysaccharide species.

### Structural Characterization of Single-Stranded Amylose and Cellulose

We first assessed the structural change in each of the single-stranded systems, in terms of the RMSD, i.e., the difference from their reference configurations. The results of this analysis are shown in the Supporting Information, Figure S1. This analysis revealed that the amylose chains behaved differently using the CHARMM36 and Drude FFs. The CHARMM simulations displayed relatively stable RMSD values throughout the 1-μs trajectories, but frequent transient spikes indicated some conformational variability in the amylose chains. On the other hand, the Drude simulations showed more dynamic amylose behavior, indicating increased conformational dynamics and enhanced flexibility relative to the additive FF. Moreover, there was considerable variability across the Drude replicate simulations, emphasizing the importance of running multiple independent simulations to ensure robust sampling of the conformational space and to assess the variability in the observed dynamics.

To further characterize the conformational variability suggested by the RMSD analysis, clustering was performed on the trajectories using MDANCE, an efficient clustering package for identifying dominant conformational states based on RMSD metrics.^62^ The CHARMM simulations of amylose resulted in an optimal result of 13 clusters based on the DB index (Supporting Information, Figure S2), with the top four clusters each accounting for approximately 10–12% of the total frames (Supporting Information, Figure S3). The top four clusters shared a common feature: extended helical conformations with a longer helical pitch compared to the initial structure (Figure 2A and Supporting Information, Figure S4). In contrast, the Drude simulation produced 28 clusters, with the largest cluster containing only ∼8% of the total frames, and 25 of these clusters contributing less than 5% (Supporting Information, Figure S3). This distribution from Drude simulations of amylose reflects a more diverse conformational ensemble. Only the most populated Drude cluster retained a semihelical structure (Figure 2B), whereas the second and third clusters with the highest population adopted compact conformations, in which the ends of the amylose chain were closer together (Supporting Information, Figure S5). These findings are consistent with the broader conformational sampling observed in the RMSD profiles. Overall, the RMSD analysis and the subsequent clustering suggest that the inclusion of polarization effects leads to a broader exploration of the conformational landscape of amylose compared to the CHARMM FF. This increased plasticity may be related to the explicit treatment of electronic polarization in the Drude model, which could allow for more sensitive interactions within the system.

**Figure 2:**
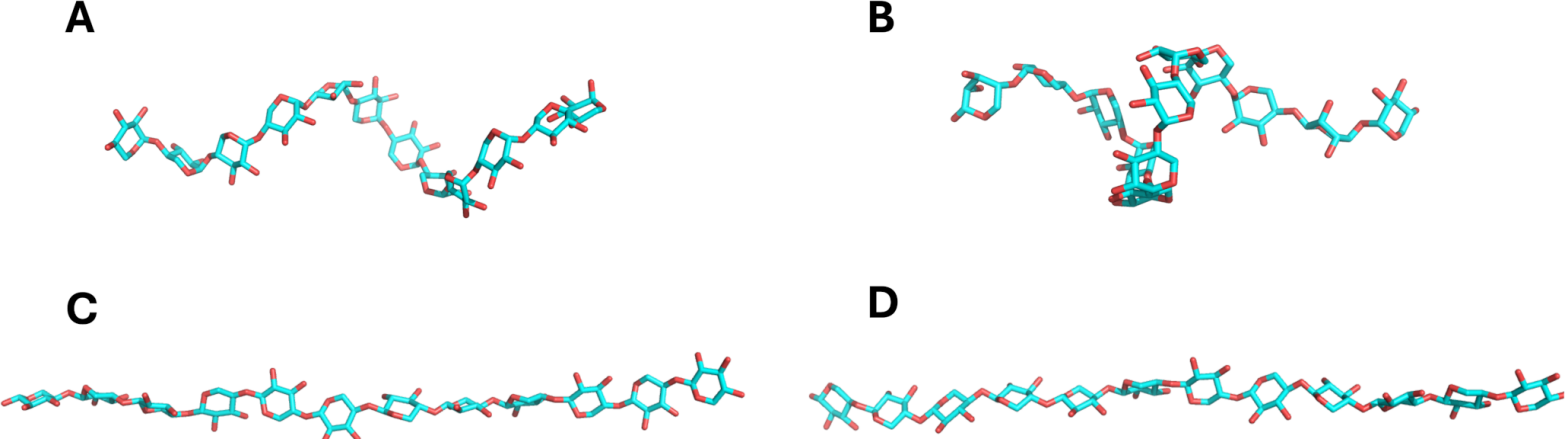
Representative structures of all clusters identified for amylose and cellulose simulations. (A) Amylose with the CHARMM FF, (B) amylose with the Drude FF, (C) cellulose with the CHARMM FF, and (D) cellulose with the Drude FF.

In the case of cellulose, the RMSD values produced by both the CHARMM and Drude FFs were more consistent than in the case of amylose (Supporting Information, Figure S6). Across all three replicates, the cellulose systems, whether simulated with CHARMM or Drude, maintained relatively low RMSD values throughout the 1-μs simulations, with only transient increases. For both CHARMM and Drude simulations of cellulose, the average RMSD was 4 ± 1 Å. In contrast, the CHARMM simulations of amylose yielded an average RMSD of 7 ± 2 Å and the Drude simulations showed even higher value of 11 ± 3 Å. This result suggests that cellulose, irrespective of the FF used, retains a narrower conformational ensemble than amylose. The higher RMSD values observed for amylose reflect its greater conformational variability in dilute aqueous solution. That is, the α(1→4) linkages introduce flexibility that allows the chain to adopt diverse coil-like geometries. In contrast, the β(1→4) linkages in cellulose impose geometric constraints and promote stabilizing intrachain hydrogen bonding, limiting structural drift. Thus, even when modeled as single short oligomers, amylose exhibits the expected flexibility while cellulose retains its characteristic rigidity. This comparison provides a controlled benchmark for evaluating how CHARMM and Drude FF capture intrinsic conformational behavior. Consistent with the RMSD analysis, clustering of the cellulose trajectories with MDANCE revealed a narrower conformational landscape for both FFs. The clustering detected two distinct clusters for each FF, with similar occupancies. The top cluster from the CHARMM simulations contained 69% of total frames and that of the Drude simulations contained 65% of the frames. The representative structures from the CHARMM and Drude simulations are shown in the Supporting Information, Figures S7 and S8, respectively, and showed elongated conformations (Figure 2C,D). These findings further support the conclusion that cellulose maintains a relatively narrow conformational ensemble, with minimal differences between the CHARMM and Drude FFs. This outcome agrees with earlier simulations of 9-unit cellulose oligomers, which likewise reported elongated conformations throughout the trajectory.^44^

Root-mean-square fluctuation (RMSF) measures the flexibility of individual atoms or functional groups, offering valuable insights into the local dynamics of the molecule.^63^ RMSF plots for unrestrained amylose in the simulations using CHARMM and Drude FFs are shown in Figure 3A, showing fluctuation levels of all 12 glucose residues. These values were calculated by averaging across the three replicate simulations. There is a clear difference in flexibility between the two FFs, with the Drude FF exhibiting systematically greater fluctuation along the entire amylose chain than the CHARMM FF. Both CHARMM and Drude systems showed the expected increase in fluctuation at the terminal residues, indicative of increased flexibility at the termini of the polysaccharide chains. Conversely, residues near the middle of the amylose chain were more rigid. However, even among these central residues, the Drude simulations consistently produced slightly greater RMSF values than the CHARMM simulations. Also, the standard deviations shown by error bars on the Drude points are larger than those for CHARMM, reflecting a greater heterogeneity in the conformational sampling with the polarizable Drude model. These observations suggest that the Drude FF allows for a greater range of conformational flexibility in amylose than CHARMM, likely due to the explicit treatment of polarization effects.

**Figure 3:**
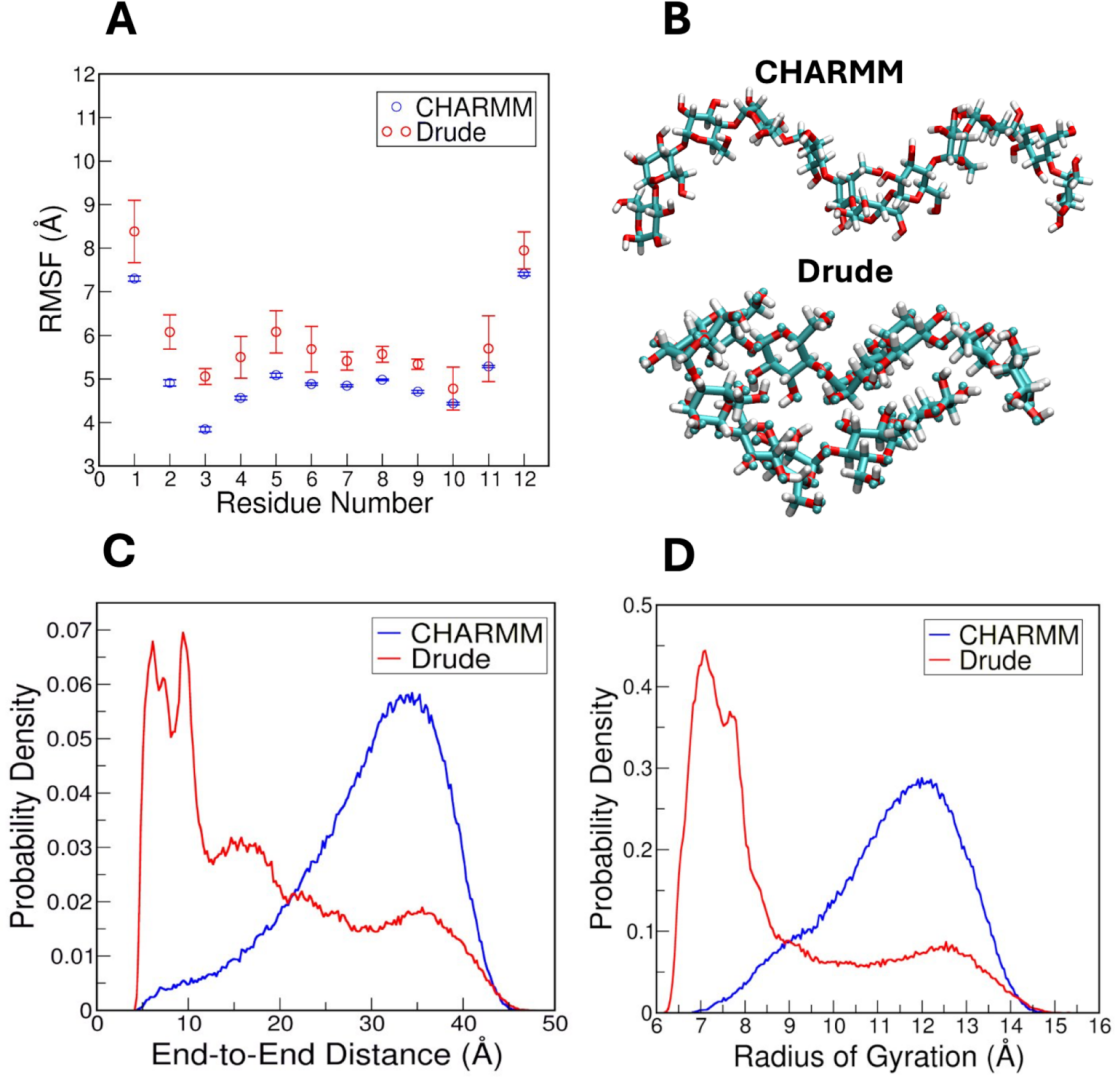
Structural analyses and their comparison between CHARMM and Drude FFs for unrestrained amylose. (A) Per-residue RMSF of the amylose chain. (B) An extended conformation of amylose chain in CHARMM and a more compact conformation of amylose in Drude system. Probability densities of the (C) end-to-end distance and (D) the radius of gyration, respectively.

End-to-end distance (*R*_ee_) is calculated as the separation between the two termini of a polymer chain.^64^ We calculated the evolution of the average *R*_ee_ over three replicates of CHARMM and Drude simulations. The CHARMM FF yielded systematically greater average *R*_ee_ values compared to those of the Drude FF over the simulation time (Supporting Information, Figure S9A). Additionally, the *R*_ee_ probability density (Figure 3C) shows that although simulations using CHARMM and Drude FFs sampled the same range of *R*_ee_, the CHARMM FF favored values around 35 Å, whereas the Drude FF featured a dominant peak near 10 Å. This difference indicates that the Drude FF samples more collapsed states of amylose, compared to the more extended conformations produced by the CHARMM FF (Figure 3B). This result further contextualizes the clustering results discussed above (Supporting Information, Figures S4 and S5), in that the collapsed structures produced by the Drude FF were disordered with proximal termini rather than being coiled in helical-like structures. The imperfect helices and collapsed states we observed align with previous MD analyses demonstrating that amylose helices are destabilized by band-flips and kinks in aqueous solution, yielding irregular hydrogen-bonding networks rather than perfectly ordered structures.^48^

The radius of gyration (*R*_g_) assesses the three-dimensional compactness of a molecule, representing the root-mean-square distance of its atoms from the center-of-mass.^65^ As with our findings on *R*_ee_ for the unrestrained amylose, the evolution of *R*_g_ (Supporting Information, Figure S9B) and its probability density (Figure 3D) show the same range of *R*_g_ values for CHARMM and Drude systems. However, their probability densities demonstrate that the Drude model exhibits a sharper and higher peak at a lower *R*_g_ value compared to the CHARMM model. This result implies that the Drude model predominantly adopted more compact conformations. In contrast, the distribution of *R*_g_ in CHARMM system is more diffuse with lower peak in the probability density graph (Figure 3D), reflecting a wider range of amylose conformations throughout the simulations. The similarity in the probability density graphs of the *R*_g_ and *R*_ee_ suggests that the observed compaction of amylose in the Drude simulations is primarily due to the two terminal ends of the chain approaching each other, rather than compaction of inner residues. We performed the same analysis on the cellulose simulations to assess the consistency of conformational behavior across FFs. As shown in Figure 4A, RMSF calculations of the single cellulose chain in the CHARMM and Drude simulations reveals nearly identical flexibility profiles in both CHARMM and Drude simulations. The terminal residues of the cellulose chain were more flexible than the residues in the middle of the chain, which is an expected outcome due to their exposure and lack of neighboring interactions. The *R*_ee_ of the cellulose chain in CHARMM and Drude systems were essentially indistinguishable (Figure 4B and Supporting Information, Figure S10A) indicating that both FFs capture a comparable degree of extension along the chain. The same holds true for the *R*_g_, which also showed minimal differences between the two simulations (Figure 4C and Supporting Information, Figure S10B).

**Figure 4:**
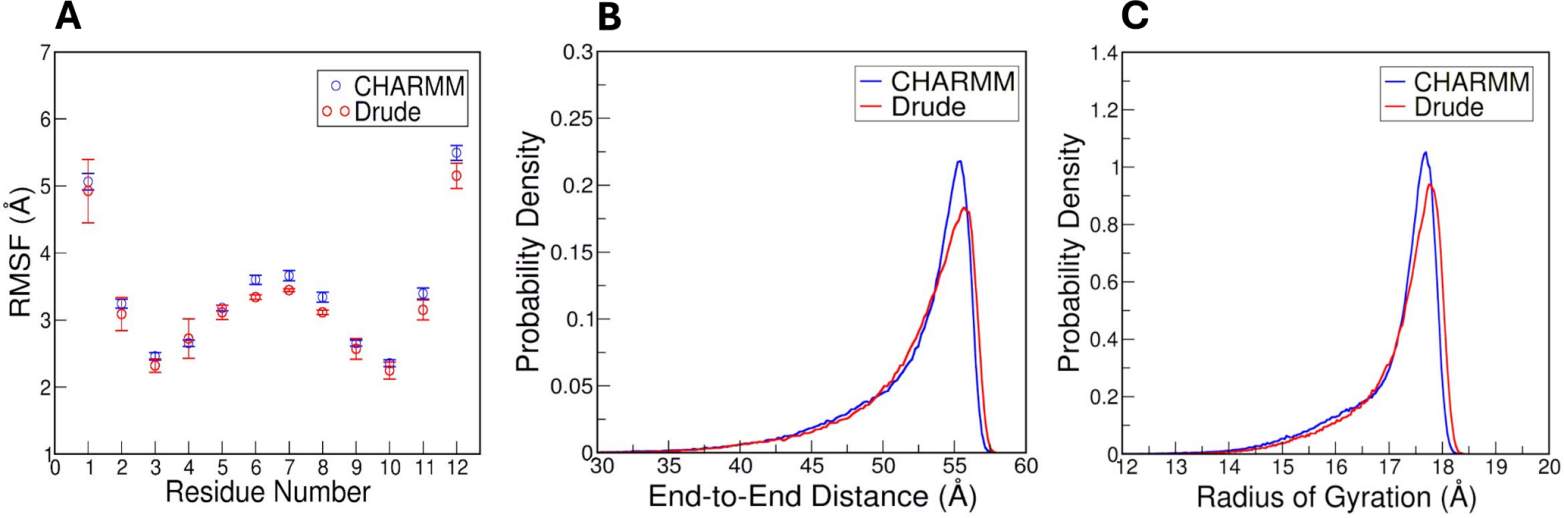
Structural analyses and their comparison between CHARMM and Drude FFs for cellulose. (A) Per-residue RMSF of the cellulose chain. Probability densities of the (B) *R*_ee_ and (C) the *R*_g_, respectively.

Amylose and cellulose likely respond very differently in the Drude polarizable FF because of their distinct chemistries. Amylose, an α(1→4) glucan, is relatively flexible and forms helix-like coils in water with only “imperfect” intrachain hydrogen bonding.^48^ Its helical cavity is largely hydrophobic and encourages helix formation and internal packing,^66^ so when electronic polarization is included, induced dipoles strengthen the effective attractions among the glucose units. As a result, our Drude simulations showed amylose folding into tighter conformations—manifesting as a lower *R*_g_ and shorter *R*_ee_ than in the corresponding CHARMM simulations. In contrast, cellulose is a β(1→4) glucan whose chain is intrinsically stiff and equipped with equatorial −OH groups that form a regular pattern of intrachain hydrogen bonds.^67^ As such, even with explicit electronic polarization, the balance of forces does not change substantially. Indeed, the probability densities of RMSD, *R*_g_, and *R*_ee_ for cellulose are essentially the same with the Drude and CHARMM FFs, and clustering yielded similar (extended) conformations with both FFs. In short, the intrinsic flexibility and hydrophobic character of amylose make its conformational ensemble more sensitive to the induced-dipole forces in the Drude model and thus it collapses to a more compact form. In contrast, the rigidity from intrachain hydrogen bonding in cellulose restricts its shape so that polarization effects make little difference in its observed properties. Therefore, use of a polarizable model when simulating amylose and its binding to hydrophobic compounds may be more appropriate.

The overall conformation of an oligosaccharide is primarily influenced by the Φ and Ψ orientations of its torsion angles between glycosyl residues (Figure 5A).^13,68^ Specifically, the conformational sampling of the acetal linkages are described by the rotation around the C1–O (Φ angle) and O–C4 (Ψ angle) bonds.^69^ Characterizing these glycosidic torsion angles is essential for describing the structural flexibility and functional properties of oligosaccharides in biological systems. We calculated the Φ and Ψ values for the linkages between all 12 sugar units in unrestrained amylose and cellulose chains under both FFs (Supporting Information, Figure S11A–D). In the case of amylose, the probability density of Φ and Ψ angles reveal distinct conformational preferences in each FF. Both CHARMM and Drude models show a dominant peak around Φ = 100°. In contrast, the Ψ distribution in CHARMM is centered at −142°, whereas Drude samples a wider range around −135°. In addition, both force fields sampled secondary Ψ states, with CHARMM showing a peak near 80° and Drude near 75°. The broader range of Ψ values observed in the Drude simulations suggests greater flexibility around the glycosidic linkages and, consequently, increased conformational flexibility of amylose under the Drude FF. This observation is consistent with our clustering analysis, which revealed greater conformational variability in amylose with the Drude FF compared to CHARMM. To further illustrate these differences, we constructed two-dimensional free-energy surfaces of the Φ and Ψ torsions for both force fields (Figure 5B–E). These plots reveal two major basins in each force field, with one basin occupying a larger area and corresponding to lower free-energy values. In the Drude model, both basins are noticeably broader than CHARMM, indicating enhanced conformational sampling and increased flexibility. The dominant basin in CHARMM is defined near (100°, −142°), whereas in Drude it spans a wider region centered near (100°, −135°), with an additional shallow minimum near (100°, 75°). These differences underscore the impact of explicit polarization in the Drude model, which allows amylose to access a more diverse set of low-energy conformations.

**Figure 5:**
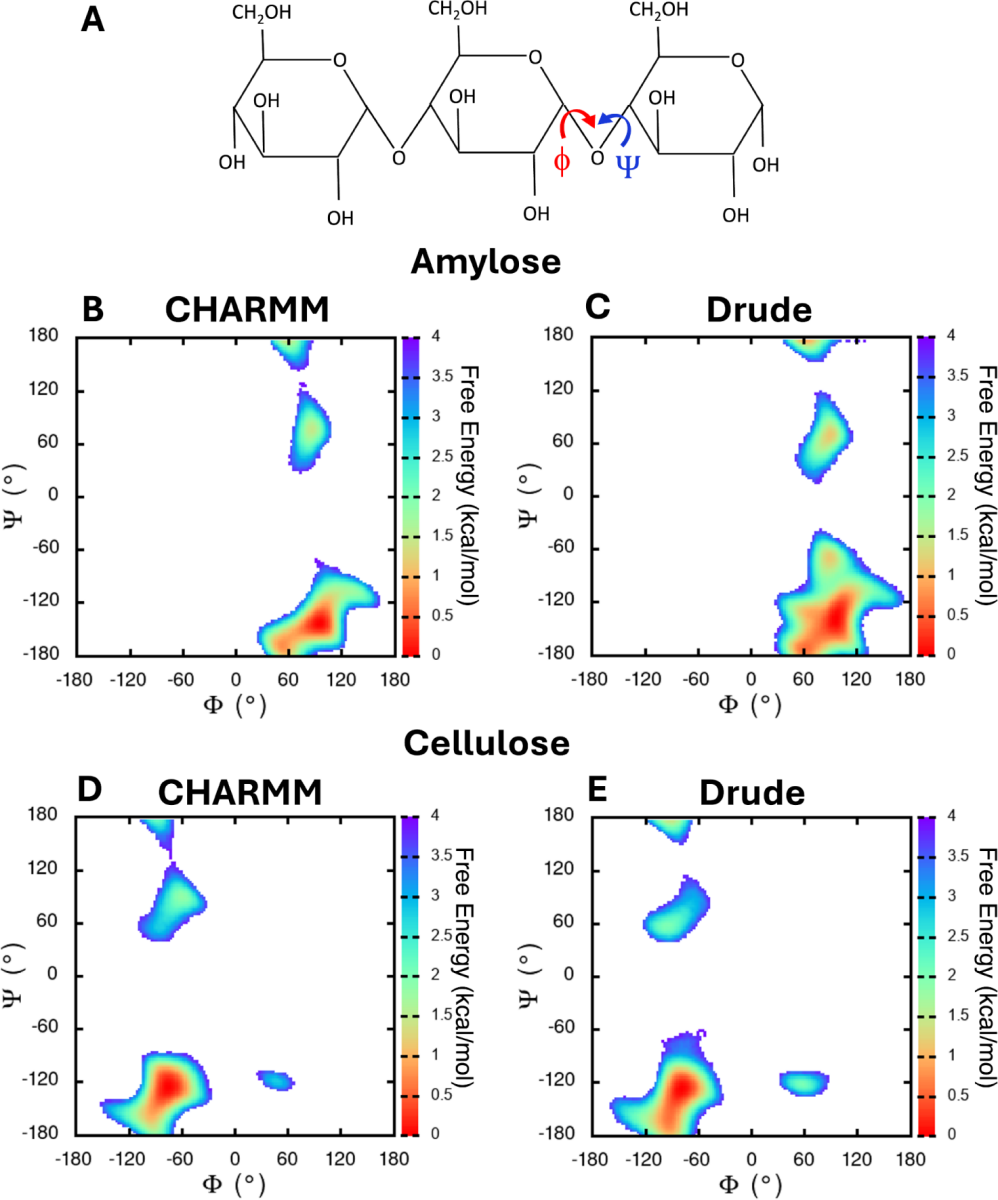
Characterization of glycosidic torsion angles in amylose and cellulose. (A) Schematic of the Φ and Ψ angles around glycosidic bonds for a polysaccharide. Free energy surfaces of of Φ and Ψ for unrestrained amylose in (B) CHARMM and (C) Drude. Free energy surfaces of Φ and Ψ for cellulose in (D) CHARMM and (E) Drude.

For cellulose, the probability density plots of dihedral angles (Supporting Information, Figure S11C–D) show that the Φ distributions are closely aligned with both force fields exhibiting a dominant peak around −80°. For the Ψ angle, the distributions for CHARMM and Drude are largely centered around −125°; however, CHARMM shows a more sharply defined peak at this value and a secondary peak around ∼90°, whereas in the Drude simulation this secondary population is shifted toward values closer to 60°. The two-dimensional Φ–Ψ free-energy surfaces (Figure 5D–E) further illustrate the similarity between the CHARMM and Drude force fields for cellulose. Both maps show the same major low-energy basin centered around Φ ≈ −80° and Ψ ≈ −125°, consistent with the dominant populations observed in the probability distributions. A secondary, higher-energy basin appears at positive Ψ values for both models, though CHARMM displays a slightly more localized minimum compared to the broader, more diffuse basin observed with Drude. Overall, the location and shape of the free-energy minima are highly comparable between CHARMM and Drude, reinforcing that both force fields stabilize similar glycosidic conformational ensembles for cellulose.

Another interesting aspect of the dynamics of these polysaccharides is sugar puckering. We used the Cremer-Pople convention^70^ to analyze and quantify the deviations from planarity in each of the glucopyranose rings. In the Cremer-Pople method, the puckering geometry with respect to a mean plane is described by θ and φ parameters. The θ parameter is the polar angle within a spherical coordinate system and indicates the general type of puckering conformation such as chair or boat. The φ parameter serves as the phase angle, and it defines the specific type of puckering deformation that occurs within the ring. It captures the nature of the puckering, such as whether the puckering forms a boat conformation, twist-boat conformation, or other typical conformations observed within cyclic compounds. The important ^4^ *C*_1_ or ^1^ *C*_4_ chair conformations are indicated by θ = 0° and θ = 180°, respectively. When θ = 90°, the ring samples boat (φ = 0, 60, 120, 180, 240, or 300°) or twist-boat conformations (φ = 30, 90, 150, 210, 270, or 330°).

With the CHARMM FF, all rings in amylose exhibited θ values near 0°, indicating a preference for the ^4^ *C*_1_ chair conformation (Figure 6A). With the Drude FF, while predominantly sampling the ^4^ *C*_1_ chair conformation, amylose also sampled the ^1^ *C*_4_ chair and boat conformations (Figure 6A). The sampling of both ^4^ *C*_1_ and ^1^ *C*_4_ conformations in the Drude simulations reveals greater flexibility of ring structures compared to the CHARMM simulations. Experimentally, it has been demonstrated that the ^4^ *C*_1_ chair-puckering conformation is favored.^49^ This conclusion was also reached in another comparative study by Chytra et al. on the CHARMM and Drude FFs in the context of α-d-glucose monosaccharides.^71^ Their work highlighted differential conformational preferences using CHARMM and Drude FFs. Specifically, the analysis determined that simulations with the CHARMM FF samples predominantly the ^4^ *C*_1_ conformation but simulations with the Drude FF exhibited greater exploration of midconformations and the ^1^ *C*_4_ conformation.^71^ Our results confirm that these puckering dynamics extend to polysaccharide systems and emphasize the conformational flexibility that exists with the Drude FF, which may arise due to lower energy barriers to structural interconversion. Importantly, experimental evidence also suggests that pyranoses such as glucose and GlcNAc are not exclusively rigid ^4^ *C*_1_ chairs, but can transiently access nonchair geometries. Atomic force microscopy studies of glucose polymers inferred the presence of non-^4^ *C*_1_ conformations.^72–74^ Thus, the broader sampling observed with the Drude FF is consistent with experimentally supported flexibility, even though the ^4^ *C*_1_ chair remains the predominant conformation.

**Figure 6:**
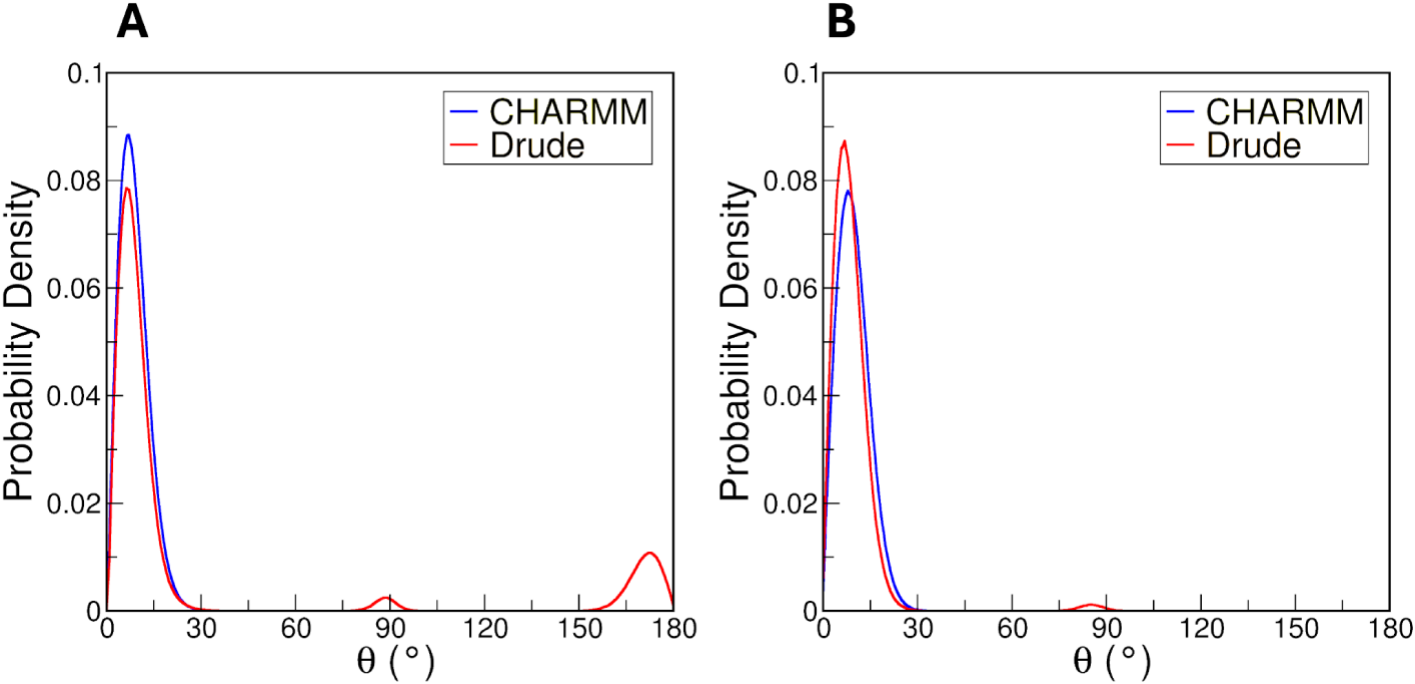
Probability densities of θ values using the Cremer-Pople convention for (A) amylose and (B) cellulose chains.

We also calculated the θ values for the cellulose chain in the CHARMM and Drude systems (Figure 6B). The probability densities of θ values in both FFs exhibited a prominent peak near 0° and the Drude FF produced a very small population at 90°. Thus, the conformational plasticity with the Drude FF persists for cellulose, indicated by the presence of boat and twist-boat conformations, but to a smaller degree than in the case of amylose. Nevertheless, both the CHARMM and Drude FFs consistently favor the ^4^ *C*_1_ chair conformation, the expected geometry for this system.

### Hydration of Single-Stranded Amylose and Cellulose

The radial distribution function (RDF) provides valuable insights into the spatial distribution of particles around a reference particle, shedding light on liquid structure, and thus the intermolecular interactions within a system. In aqueous solution, hydrogen bonds, are critically important for determining the structure of water and its interactions with solutes. We calculated the RDF for water around each of the oxygen atoms within sugar units in both CHARMM and Drude FF systems and analyzed the total number of intramolecular hydrogen bonds within the polysaccharide chain as well as those formed between the chain and water molecules for all unrestrained and restrained amylose systems.

The resulting RDF plots for unrestrained amylose reveal distinctive hydration characteristics between the CHARMM and Drude systems. Notably, in both FFs, hydroxyl oxygen atoms O2, O3, and O6 were more hydrated than O4 and O5, the atoms involved in the glycosidic linkage and functioning solely as hydrogen bond acceptors (Figure 7A,B). Consistently, these hydroxyl groups also formed a greater number of hydrogen bonds with water molecules (Figure 8C,D) as a result of their solvent accessibility and their dual role as hydrogen bond donors and acceptors. Our findings align well with the study by Khatami et al.,^48^ who used MD simulations to investigate hydrogen bonding patterns of single amylose chains in water. They found that amylose chains formed a greater number of hydrogen bonds with water molecules at the O2, O3, and O6 positions. Additionally, their study highlights the dynamic nature of hydrogen bond formation and breakage, aligning with the fluctuating behavior we observed in different FFs.^48^

**Figure 7:**
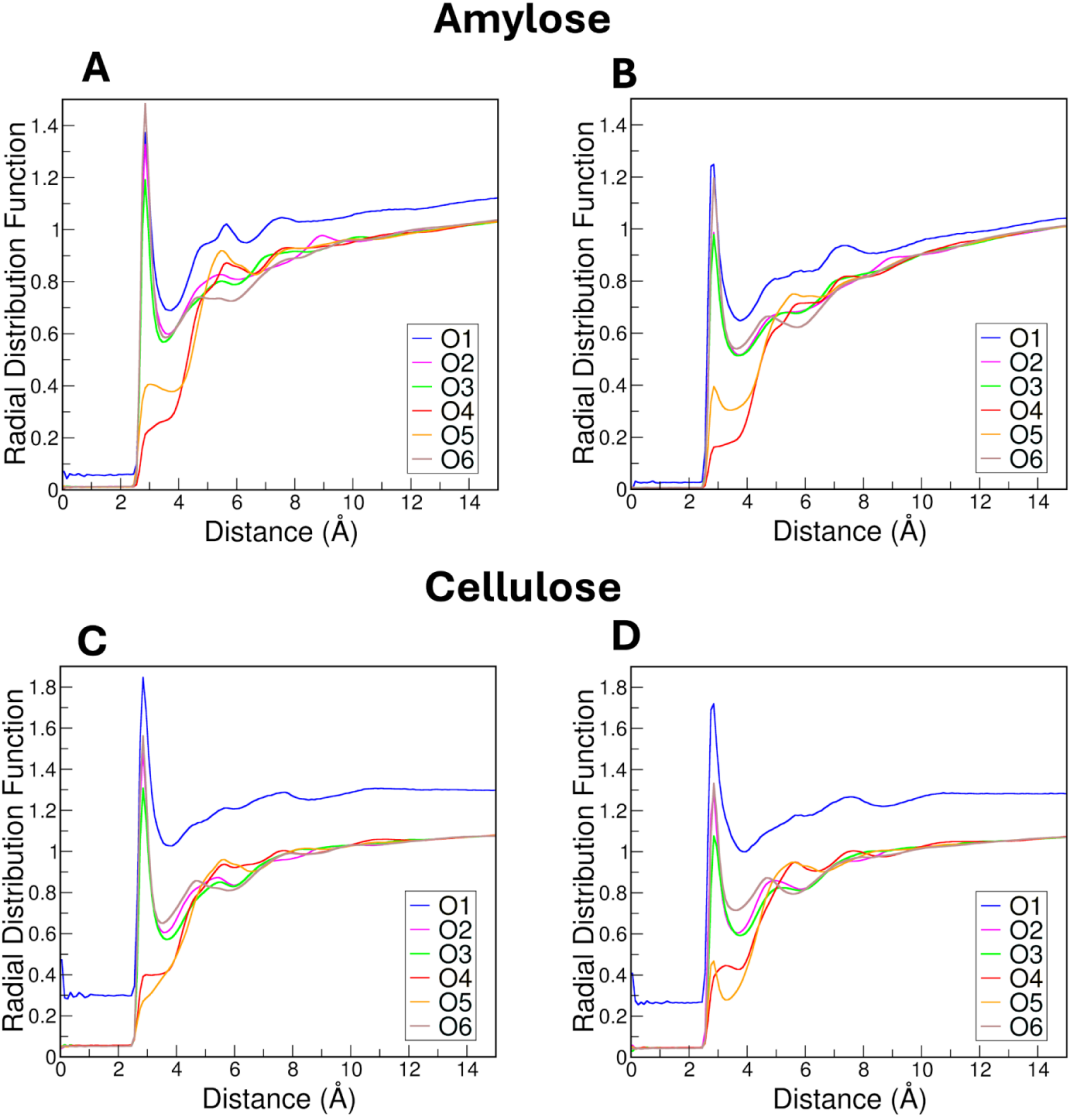
Radial distribution functions for unrestrained amylose and cellulose. Amylose RDFs are shown for the CHARMM and Drude FFs in panels (A) and (B), respectively. Cellulose RDFs with the CHARMM and Drude FFs are shown in panels (C) and (D), respectively.

**Figure 8:**
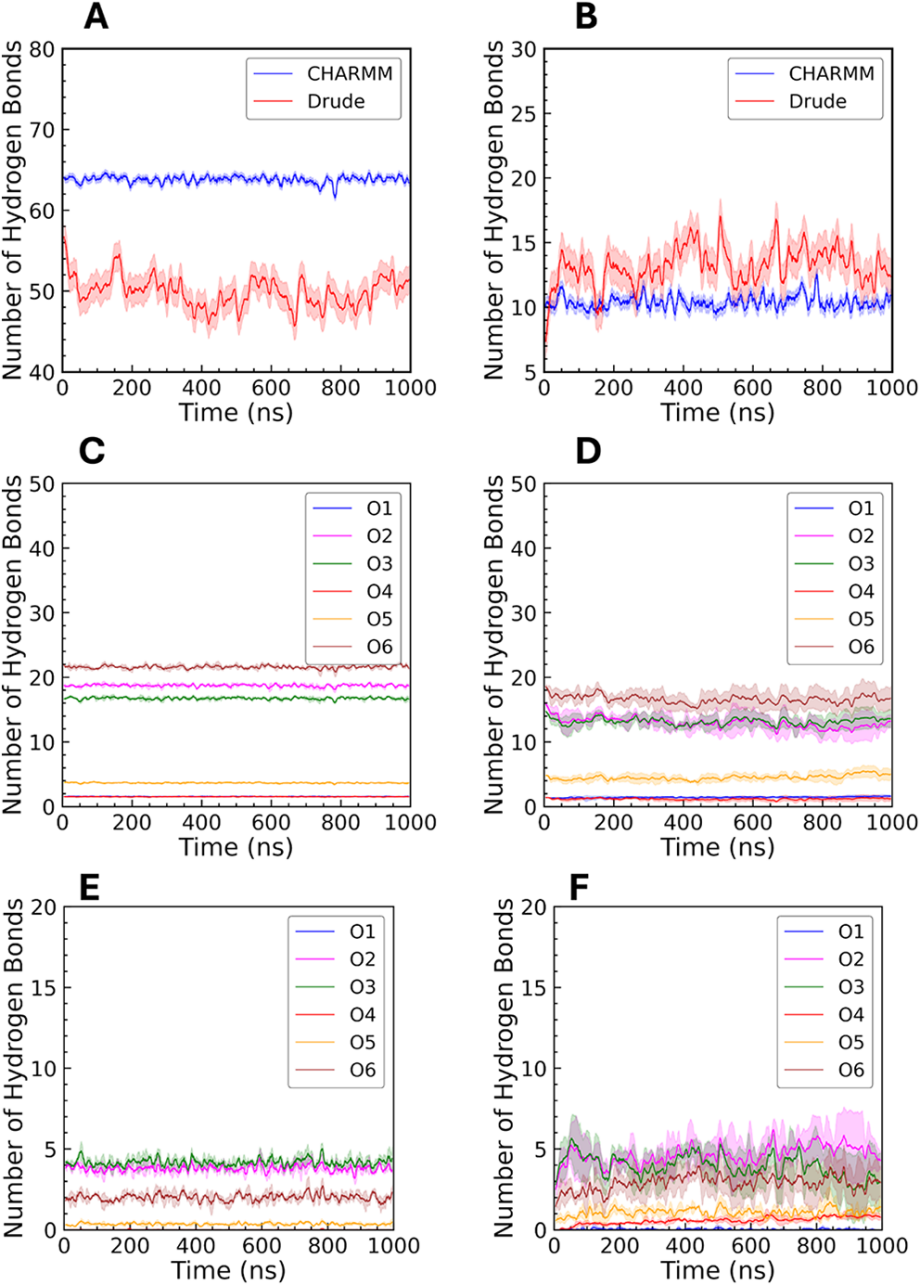
Hydrogen bonding in CHARMM and Drude systems for unrestrained amylose. Total number of (A) amylose-water (intermolecular) and (B) amylose-amylose (intramolecular) hydrogen bonds with both CHARMM and Drude FFs. Panels (C) and (D) show the number of hydrogen bonds formed between each of the oxygen atoms in the sugar rings and water in the CHARMM and Drude simulations, respectively. Panels (E) and (F) show the number of polysaccharide intramolecular hydrogen bonds in the CHARMM and Drude simulations, respectively.

Within the CHARMM system, the oxygen atoms O2, O3, and O6 exhibited much greater hydration (Figure 7A,B) and a greater number of hydrogen bonds with water (Figure 8C,D) compared to their counterparts in the Drude system, suggesting a stronger attraction of water molecules to these hydroxyl groups. These hydrogen bonds were relatively consistent throughout the simulation, reflecting stable hydration of hydroxyl groups. In contrast, the Drude FF produced more intramolecular hydrogen bonds (Figure 8B), and these interactions fluctuated more frequently during the simulations, indicating dynamic exchange events. Figure 8E,F illustrate the intramolecular hydrogen bonding for individual oxygens within the amylose chain using CHARMM and Drude models. These results indicate that the CHARMM model tends to maintain stable intramolecular hydrogen bonds, contributing to a more rigid structure. In contrast, the Drude model’s dynamic behavior highlights greater structural flexibility, allowing the amylose chain to explore a wider range of conformations.

It is worth noting that the O1 atom, situated at the reducing end of the chain, was prominently hydrated and formed hydrogen bonds with water molecules, consistent with its exposed position allowing interaction without steric hindrance from neighboring glucose units. As expected, O4 consistently exhibited the lowest hydration peak in the RDF and the fewest hydrogen bonds with water, reflecting its role as the glycosidic ether oxygen with limited hydrogen-bonding capacity. Hydration and hydrogen-bond behavior were similar in the restrained systems of amylose (Supporting Information, Figure S12 and S13), with O2, O3, and O6 maintaining higher RDF peaks and a greater frequency of hydrogen bonds than O4 and O5. The reduced interaction of O4 and O5 with water is consistent with their ether character, in contrast to the hydroxyl oxygens that readily form hydrogen bonds with solvent.

We also calculated RDFs and hydrogen bonding patterns for individual oxygen atoms in cellulose chains (Figure 7C,D and 9). The RDF profiles and H-bond analysis yielded similar results as in the amylose chain. In CHARMM, hydroxyl oxygen atoms O2, O3, and O6 were more hydrated and formed more hydrogen bonds with water compared to O4 and O5, indicating a greater propensity for solvent interaction. For intrachain hydrogen bonds, O3 and O5 were especially prominent in cellulose, differing from amylose, where O2, O3, and O6 dominated intramolecular interactions. O4 again exhibited the lowest hydration and fewest hydrogen bonds. These results are consistent with prior studies by Shen and Gnanakaran demonstrating the stability of O3H3···O5 intrachain hydrogen bonds in cellulose.^75^

**Figure 9:**
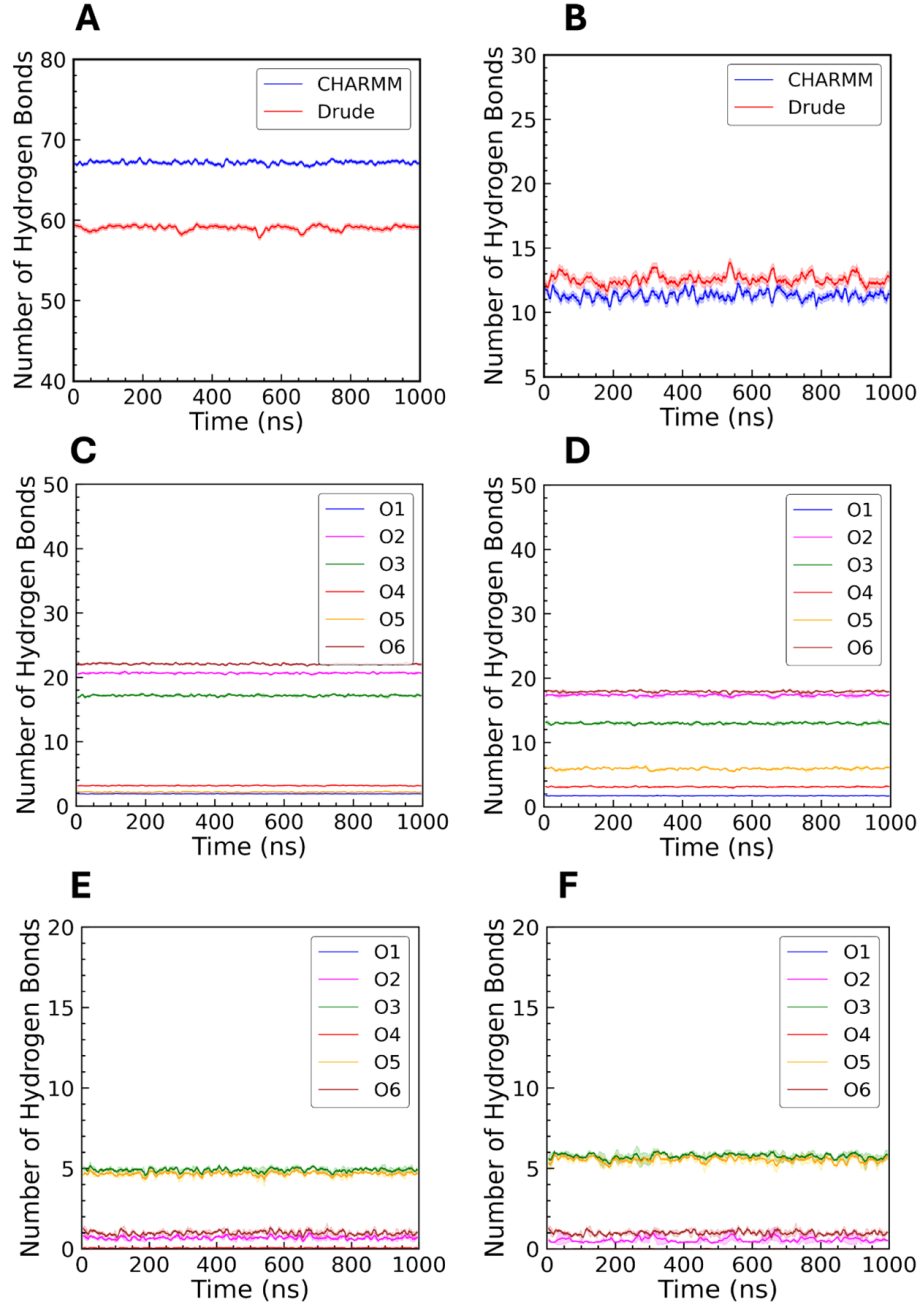
Hydrogen bonding in CHARMM and Drude systems for cellulose. Total number of (A) cellulose-water (intermolecular) and (B) cellulose-cellulose (intramolecular) hydrogen bonds with both CHARMM and Drude FFs. Panels (C) and (D) show the number of hydrogen bonds formed between each of the oxygen atoms in the sugar rings and water in the CHARMM and Drude simulations, respectively. Panels (E) and (F) show the number of intramolecular polysaccharide hydrogen bonds in the CHARMM and Drude simulations, respectively.

Taken together, the combined RDF and hydrogen-bond analysis suggests that while the hydration properties of amylose and cellulose are broadly similar, there are subtle differences as a function of inclusion of electronic polarization in the simulation. CHARMM simulations indicated a greater number of water molecules around hydroxyl groups and more stable hydrogen bonds with water, whereas Drude simulations favored intramolecular hydrogen bonding and exhibited more dynamic exchange. This difference may be explained by the anisotropic treatment of electronic polarization and the inclusion of “lone pair” virtual sites on oxygen atoms in the Drude FF, which reflect the directionality of electrostatics intrinsic to these functional groups and manifest as slightly reduced hydration compared to the additive CHARMM FF.

### Electrostatic Properties of Single-Stranded Amylose and Cellulose and Hydrating Waters

Figure 10 illustrates the probability densities of dipole moments for the sugar rings in amylose and cellulose chains using CHARMM and Drude polarizable FFs. Given the flexibility of the sugar rings (puckering) and rotation of hydroxyl groups, these dipole moments reflect contributions from both nuclear geometry and electronic plasticity. For both amylose and cellulose, the dipole moment distributions with the CHARMM FF are generally unimodal and have a slightly wider range compared to the values produced by the Drude FF. For amylose, the average dipole moment was 5 ± 2 D in both the CHARMM and Drude simulations. For cellulose, the CHARMM simulations yielded an average dipole moment of 4 ± 2 D, while the Drude simulations produced a value of 3 ± 1 D. This outcome indicates that the sugar rings in the CHARMM system generally possess higher dipole moments. This property can be attributed to the intrinsic mean-field charge assignment in additive FFs, which is designed to reflect average properties in an aqueous medium. Conversely, the dipole moment distributions from the Drude FF simulations are slightly narrower with sharper peaks, indicating a different, and responsive representation of dipole moments. The polarizable nature of the Drude FF captures real-time electronic fluctuations, in response to the local electrostatic environment and resulting in a dynamic hydrogen bonding network.

**Figure 10:**
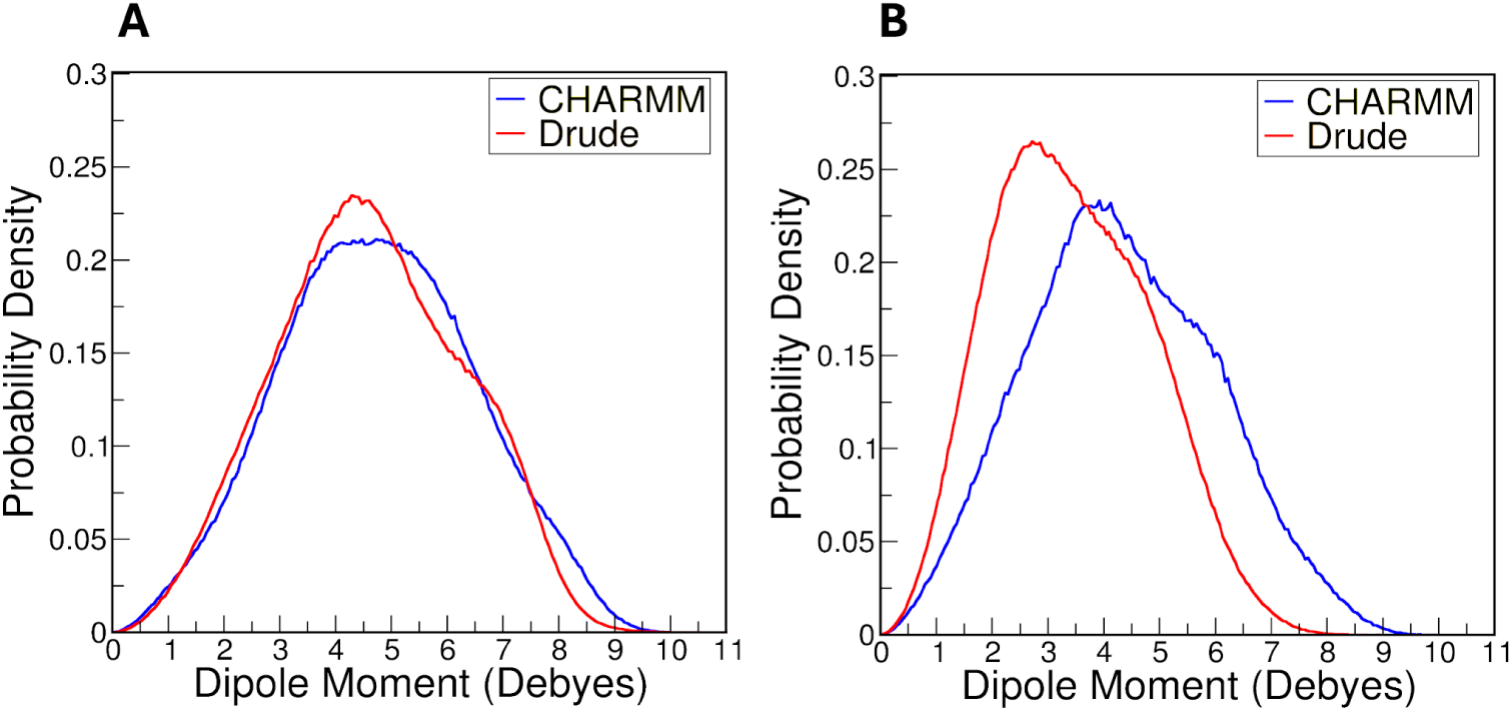
Probability densities of sugar unit dipole moments in (A) amylose and (B) cellulose.

In addition to investigating the dipole moments of constituent glucose units, it is also of interest to calculate dipole moments of water molecules that directly interact with the polysaccharides. By assessing variations in water dipole moments, we sought to quantify the influence of induced polarization by carbohydrates on the surrounding solvent structures and solvation dynamics.^76^ Our analysis focused on calculating the dipole moments of water molecules confined within the restricted chain of amylose (amylose restrained 1, which set Φ = 103° and Ψ = 115°). We selected this structure due to the presence of clearly defined cavity within its helical configuration. Prior to this analysis, we aligned the helical axis along the *x*-axis to ensure a consistent reference frame for both dipole moment and electric field vector analysis (Figure 11A). We investigated the components of the molecular dipole moments because they allow for insight into whether or not there is a preferential alignment of these molecules in the cavity.

**Figure 11:**
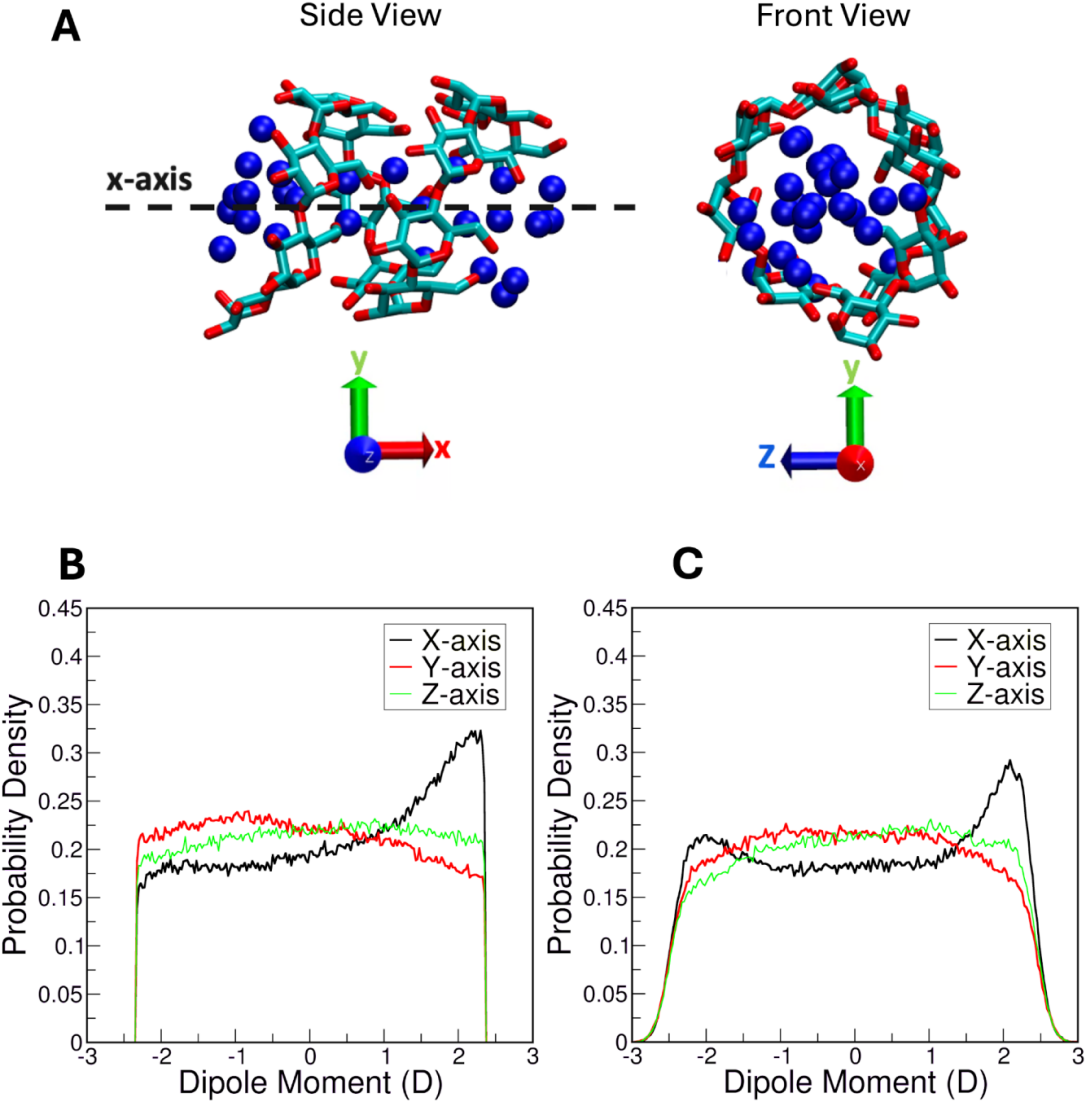
Internal hydration of amylose in a helical configuration. (A) Side and front views of the amylose helical structure aligned with *x*-axis. Blue circles show the water molecules confined inside the helix. Probability densities of water molecule dipole moment components for (B) CHARMM and (C) Drude simulations.

In simulations using the CHARMM FF, we observed a distinct peak at ∼2 D in the probability densities of water dipole moments, indicating a preference for the alignment of water dipole moments along the + *x*-axis (Figure 11B). However, the distribution displayed a near-flattened profile along the *y*- and *z*-axis, suggesting no preferential orientations along these axes. It is important to note that given the rigid geometry of the TIP3P water model, any apparent change in dipole moment here simply reflects rotational motion of the water such that it is oriented differently along the Cartesian axes. In contrast, simulations employing the Drude FF produced a bimodal distribution of water dipole moments, indicating that the waters were aligned in both directions, with some disorder in the middle (Figure 11C). In the Drude simulations, the SWM4-HLJ water model^77^ has a variable dipole moment given that it is explicitly polarizable. Across all three axes, the dipole vector components of water molecules confined within the amylose helix exhibited a range between −3 and 3 D in the Drude system, wider than that observed in CHARMM simulations. Whereas the water dipole moments from the CHARMM simulations had a hard truncation at ±2.34 D (the fixed value of TIP3P, Figure 11B), the gradual decay of these values in the Drude simulations with SWM4-HLJ (Figure 11C) reflects the impact of explicit polarization. Given the bulk dipole moment value of ∼2.58 D for SWM4-HLJ,^77^ our dipole vector component analysis suggests that along the *x*-axis, the water molecules are slightly polarized at the terminal ends of the amylose helix. The distinct peaks at the ends of the amylose helix reveal another difference between the two FFs compared here, in that the CHARMM FF (in conjunction with the TIP3P model) leads to unidirectional alignment of water molecules, while the Drude FF with the SWM4-HLJ model leads to bidirectional alignment of water. The impact of this predicted phenomenon will require additional experimental and theoretical work to validate and understand.

### Properties of the First Hydration Shell around Single-Stranded Amylose and Cellulose

The first hydration shell around a biomolecule often has different structural, dynamic, and thermodynamic properties from bulk water. In MD simulations, therefore polarizable FFs may serve as a better model of these properties.^78^ Recent work on monosaccharides supports this view, as the polarizable AMOEBA force field produces hydration shells that are denser compared to additive models, and, importantly, produces solution behavior and thermodynamic properties in closer agreement with experiment.^79^ Moreover, AMOEBA eliminates spurious sugar aggregation commonly observed with fixed-charge force fields, suggesting a better balance of intra- and intermolecular forces. We examined the dipole moments of water molecules in the first hydration shell of amylose to determine the impact of electronic polarization and to understand if the observed behavior of the AMOEBA FF is also produced by the Drude model. As in the analysis of cavity-bound water molecules, **t**he amylose chain was aligned along the *x*-axis, and dipole moments were decomposed into their *x*-, *y*-, and *z*-components.

In CHARMM simulations, the distribution of the *x*-axis dipole moment component shows a distinct peak, indicating preferential water molecule alignment along the *x*-axis in which the amylose chain is oriented (Figure 12A). Given that the distributions along the *y*- and *z*-axes are rather flat, the waters exhibited no preferred orientation in these dimensions. Additionally, as discussed above, in the CHARMM simulations, water molecules participated in a greater number of hydrogen bonds with amylose. As a result, the water molecules in the CHARMM simulations are likely somewhat restricted because they are engaged in relatively strong hydrogen bonds with surface-accessible hydroxyl groups on amylose.

**Figure 12:**
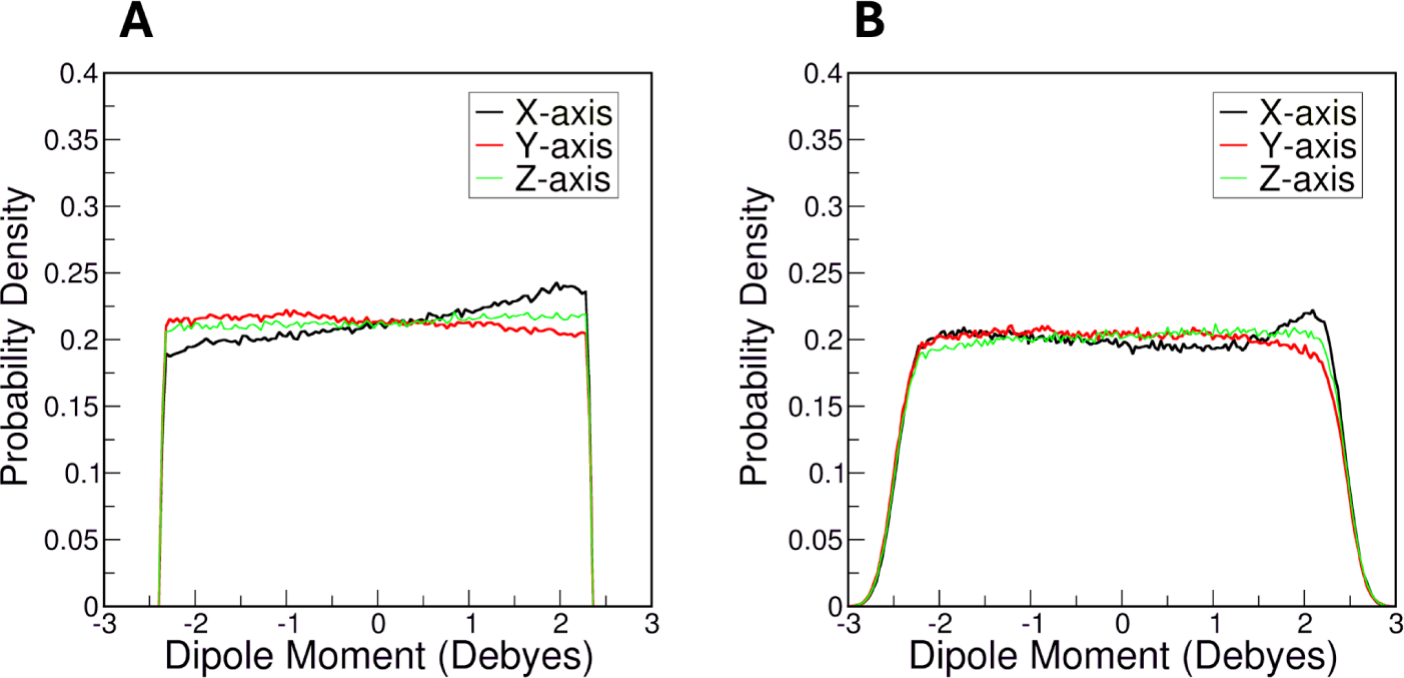
Probability densities of the dipole moments of water molecules in the first hydration shell of amylose chain in (A) CHARMM and (B) Drude simulations, respectively.

The dipole properties of first-shell water molecules in the Drude simulations were similar to those of the CHARMM simulations (Figure 12B). The *y*- and *z*-axis components of the water dipole moments were relatively flat, and along the *x*-axis there was a slight alignment effect along the +*x*-axis. As with the internal water molecules, the SWM4-HLJ water molecules manifested a wider distribution of dipole moments (from −3 to 3 D) along the three axes in the Drude simulations. Thus, we conclude that there is a subtle, but potentially important, difference in water alignment of internal water molecules in the amylose helix with the different FFs, but the first-shell water molecules behaved similarly.

### Electric Fields inside the Amylose Helix

We calculated the electric field (E⃗) exerted by the amylose chain along its axis within the helical structure using the TUPÃ software.^80^ E⃗ calculations were performed at 24 equidistant points along the axis of the amylose chain (Figure 13A). In this convention, point 1 is closest to the reducing end of the chain and point 24 is closest to the nonreducing end. E⃗ was calculated according to eq 1:

1 $${\mathrm{E⃗}}_{\left(x,y,z\right)}=\overset{N}{\underset{i=1}{\sum }}\frac{1}{4\pi \varepsilon_{0}}\times \frac{Q_{i}}{r^{2}}\times {\mathrm{r̂}}_{\left(x,y,z\right)}$$

where E⃗(*x*,*y*,*z*) is the electric field vector at the point (*x*,*y*,*z*), *N* is the number of atoms considered in the calculation, *Q*_*i*_ is the charge of atom *i*, *r* is the distance between the point of interest and atom *i*, r̂_(*x*,*y*,*z*)_ is the unit vector pointing from atom *i* to the point (*x*,*y*,*z*), and ε_0_ is the vacuum permittivity constant.^80^

**Figure 13:**
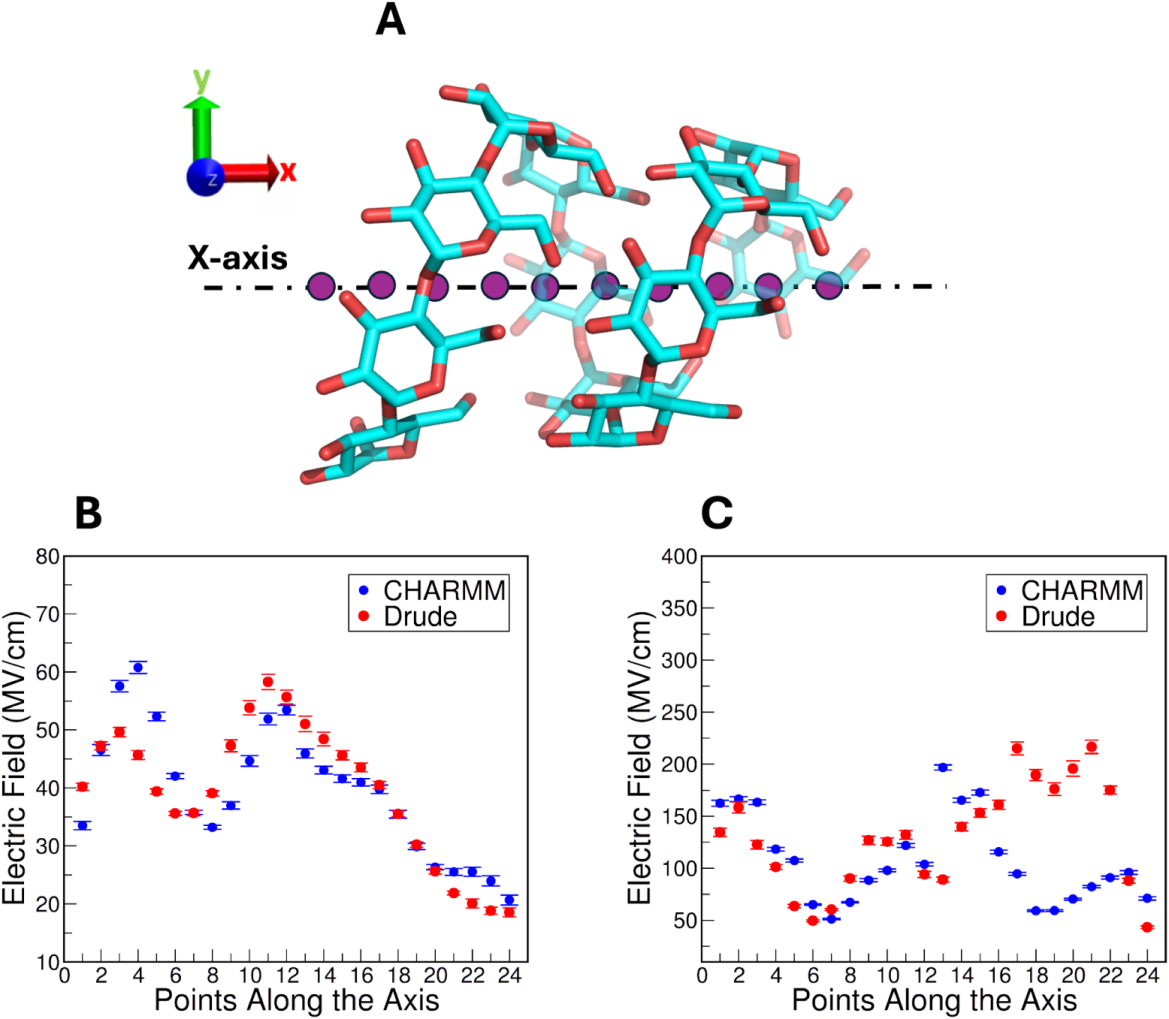
Electric field calculations along the axes of the “restrained 1” and “restrained 2” amylose structures. (A) A schematic representation of helical amylose chain and the points along the axis where E⃗ values were calculated. E⃗ values for (B) “restrained 1” and (C) “restrained 2” amylose structures. Error bars correspond to the 95% confidence interval.

The calculations were performed on the trajectories obtained from both restrained amylose systems, allowing for a comparison of the electric fields acting within a helical structure as a function of its solvent accessibility. For the single-chain system, the entire structure was divided into two blocks, each containing 6 glucose units. The dynamic coordinates of each of 24 points along the amylose chain axis were determined by calculating the minimum and maximum values of each set of 6 residues. This approach ensured that the coordinates were dynamically adjusted for each point along the helix. E⃗ values were averaged across all frames in the simulation.

E⃗ values calculated from both CHARMM and Drude “restrained 1” and “restrained 2” amylose simulations are shown in Figure 13B, C. A pronounced oscillatory pattern emerged in the E⃗ values for both FFs, signifying periodic variations in electrostatic interactions at different points along the axis. In the case of the “restrained 1” structure, which has a wider central cavity, E⃗ values with both FFs varied between 30–60 MV/cm (Figure 13B) and steadily declined to ∼20 MV/cm toward the nonreducing end of the amylose chain. Periodicity and symmetry of the E⃗ values in the CHARMM simulation of the “restrained 2” structure, which has a very narrow central cavity, were more pronounced and E⃗ values were higher than in the “restrained 1” simulation, 50–150 MV/cm (Figure 13C). Thus, it appears that compaction of the amylose structure leads to larger internal E⃗ values. This outcome is sensible given the distance-dependence of electric fields; the strength of the field decays linearly as a function of distance between the charged atom and the point in space (eq 1). In the Drude simulation of the “restrained 2” structure, some periodicity was apparent but E⃗ values were higher toward the nonreducing end of the chain than in the case of CHARMM, reaching ∼200 MV/cm before reducing to ∼50 MV/cm at the final point along the axis.

E⃗ values differed in their variation between the two FFs. The E⃗ values from the CHARMM simulation fluctuated less, leading to smaller error bars, and indicating of a less variable electrostatic environment along the amylose chain. Conversely, the E⃗ values from the Drude simulation exhibited larger error bars and greater variability, reflecting the dynamic and flexible nature of the polarizable Drude FF given the ability of induced dipoles to respond to changes in the local environment.

### Double-Stranded Amylose

In addition to investigating single-stranded amylose chains, we extended our study to explore the behavior of double-helical structures of amylose. In all the three simulation replicates using CHARMM and Drude FFs, we observed remarkable robustness in the double-stranded helical structure, which remained largely intact throughout the 1-μs simulation duration. Although transient unzipping events occurred, the helical structure promptly reformed, indicating a high degree of stability. Figure 14A illustrates the RMSD evolution of the double-helical amylose employing the CHARMM and Drude FFs. The observed stability of the double-helical structure is consistent with experimental studies reported previously. Techniques like NMR spectroscopy have confirmed the presence of a double helix structure of amylose in water, demonstrating that these duplexes are thermodynamically stable entities at sufficient chain lengths (greater than 12 glucose units) and concentrations, with stability increasing linearly with chain length and driven primarily by favorable enthalpic interactions.^81^

**Figure 14:**
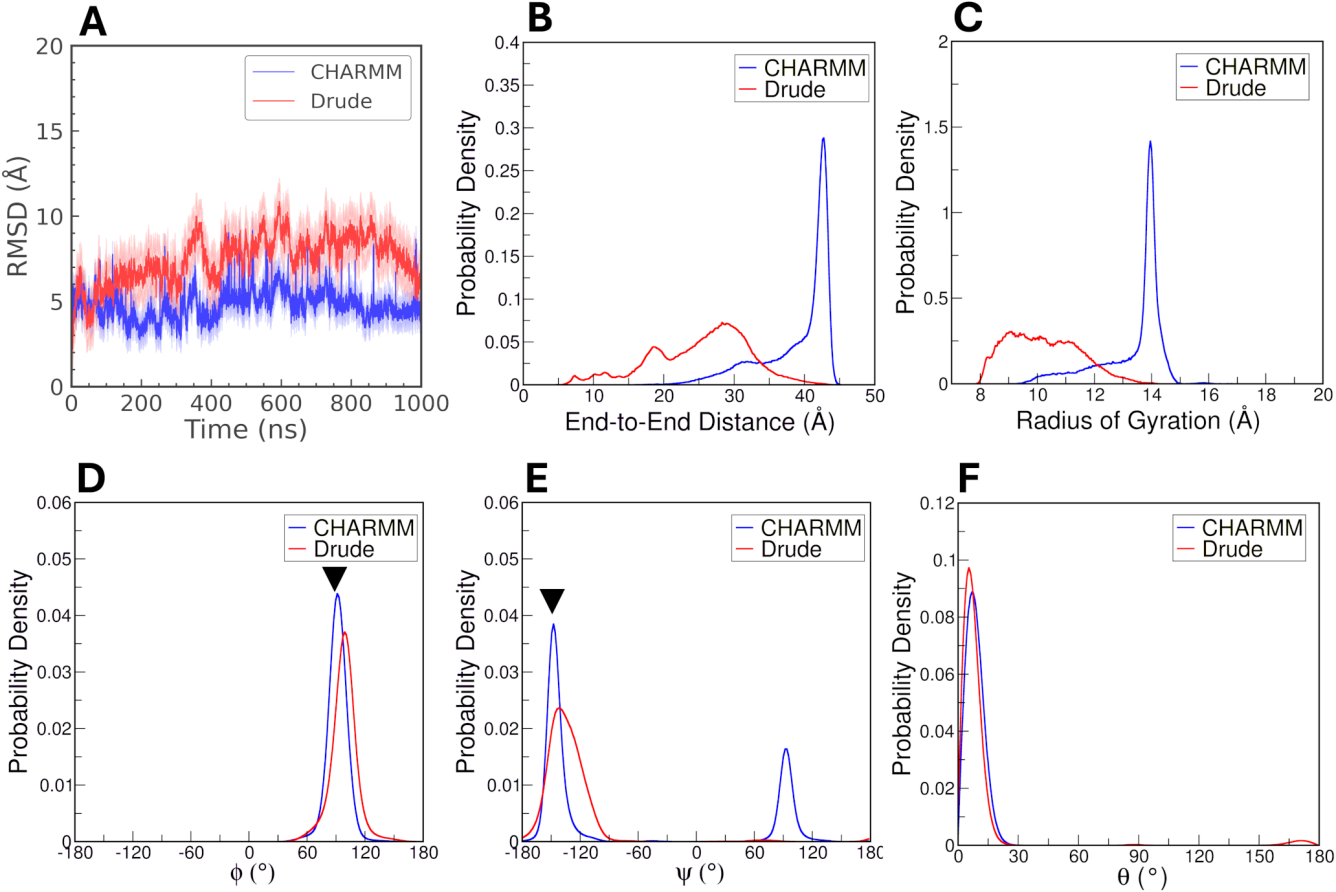
Structural characterization of the double-helical amylose structure. (A) Average RMSD over three replicates, with lighter shading indicating the standard deviation across the replicates. Probability distributions of (B) *R*_ee_, (C) *R*_g_, (D) Φ, (E) Ψ, and (F) the θ puckering parameter for CHARMM and Drude simulations. Experimental values are indicated with triangles in panels D and E.

We also computed the *R*_ee_ and *R*_g_ for the double-helical amylose for both the CHARMM and Drude FFs (Figure 14B,C). The *R*_ee_ distribution reveals that the CHARMM model exhibits a sharp peak around 43 Å, indicating a consistent and rigid double helix structure. In contrast, the Drude model shows a broader distribution, with peaks around 19 Å and 29 Å, suggesting greater variability and flexibility in the helical structure. Similarly, the *R*_g_ distribution (Figure 14C) highlights a sharp peak around 14 Å for the CHARMM model, emphasizing a stable conformation. The Drude model, however, demonstrates a broader distribution, reflecting increased flexibility and dynamic behavior. These differences underscore the CHARMM FF’s propensity for rigidity, while the Drude FF predicts a more flexible structure. The transient partial unzipping observed in Drude simulations suggests that amylose duplexes of chain length 12 are at the threshold of stability and can partially open at their termini. This observation aligns with experimental findings showing that at least 12 glucose units are required for duplex stability, which further improves with longer chains beyond a degree of polymerization of 12.^81^

We analyzed the Φ and Ψ dihedral angles for both chains of the double-helical amylose structure to assess whether the observed global structural stability was maintained at the level of glycosidic geometries. In the CHARMM simulations, we observed small fluctuations in the Φ and Ψ values throughout the simulations. Consequently, the corresponding probability densities exhibited sharper peaks compared to the Drude simulations, indicating a greater degree of structural rigidity (Figure 14D,E). The Φ sampling with the CHARMM FF aligns closely with the experimental value (Figure 14D), but for Ψ sampling, the CHARMM FF produced two peaks (Figure 14E). The dominant peak coincides with the experimental value but the second peak deviates substantially. Conversely, in the Drude simulations, both Φ and Ψ agreed well with the experimental values, though the Φ sampling deviated slightly from the experimental value.

Additionally, employing the Cremer-Pople convention,^70^ we calculated θ values to evaluate ring puckering. In the CHARMM system, θ values clustered near zero, indicating predominantly ^4^ *C*_1_ chair conformations for the constituent glucose rings. The ^4^ *C*_1_ chair conformation was also dominant in the Drude simulations, although with the polarizable model, some ^1^ *C*_4_ chair conformations were sampled (Figure 14F). This subtle shift in the distribution of θ values in the Drude simulations underscores a slight increase flexibility and susceptibility to chair conformational transitions in the Drude-modeled amylose, similar to what we observed in the case of single-chain amylose. The double-helical nature of the duplex structure likely restricts conformational fluctuations to some extent, as the emergence of ^1^ *C*_4_ chair conformations in the single-stranded amylose simulations was more pronounced (Figure 6).

Finally, we examined the hydrogen bonding between the two parallel chains as well as within each individual chain in the double helix. We observed that a greater number of hydrogen bonds formed between the two chains than within each individual chain using the CHARMM FF (Figure 15A). This observation suggests that interchain hydrogen bonding stabilizes the double-helix structure. In the Drude system, the opposite was true, such that the hydrogen bonds in a single chain were greater than those between the chains (Figure 15B). This behavior suggests weaker interchain interaction in Drude system, which can lead to the greater flexibility of the double helix that we have described above. We also compared the number of hydrogen bonds between each chain and water. The CHARMM FF produced a greater number of amylose-water hydrogen bonds than the Drude FF (Figure 15C,D). Each of these values was systematically lower than in the case of single-chain amylose with water (Figure 8A), likely due to some hydroxyl groups being engaged in interchain hydrogen bonds that stabilize the double helix. Finally, we counted the hydrogen bonds formed between the amylose chain and water molecules as a function of each hydroxyl group (Supporting Information, Figure S14). With the CHARMM FF, O2 and O3 formed a greater number of hydrogen bonds with water molecules than with the Drude FF, whereas O6 formed an equal number of hydrogen bonds with both the CHARMM and Drude FFs. These findings reflect the impact of FF selection on the balance of intermolecular interactions and stability of biomolecular structures. As in the single-chain amylose systems, the properties of double-helical amylose appear to be subtly dependent on softer interactions with the Drude FF compared to the additive CHARMM FF.

**Figure 15:**
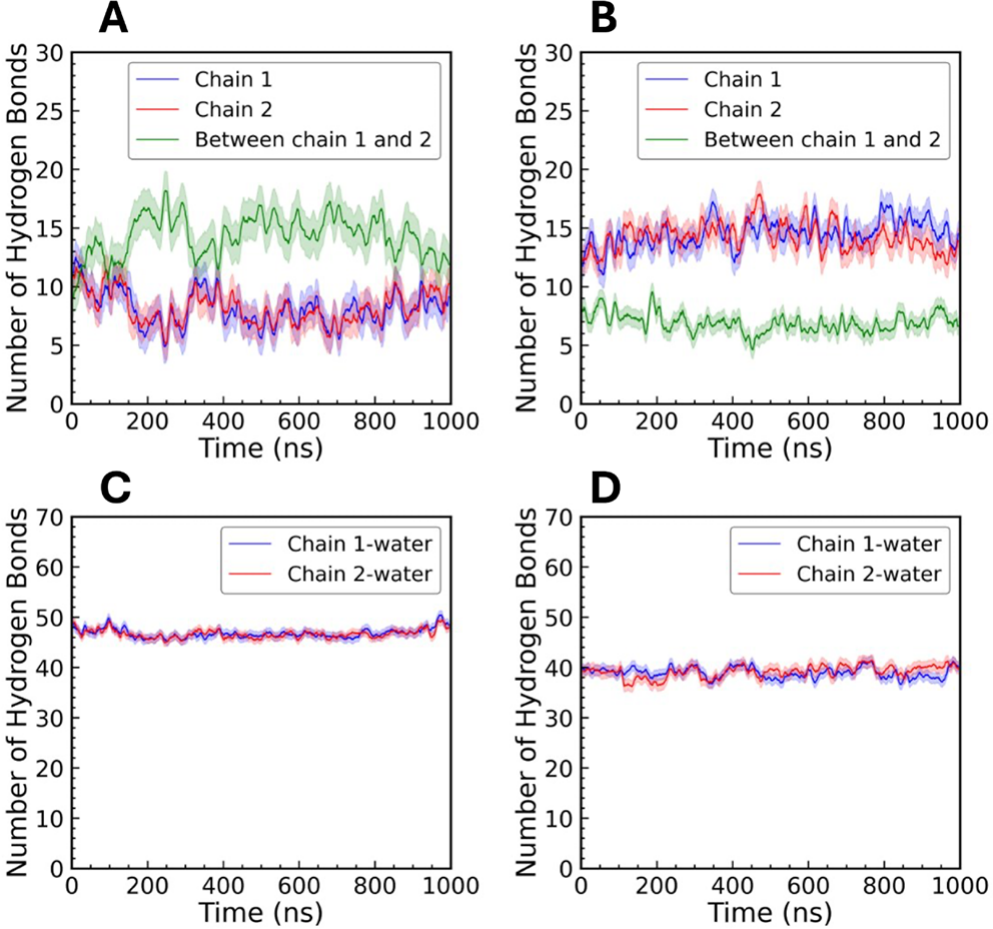
Intermolecular and intramolecular hydrogen bonding in the double-helical amylose system. The number of intermolecular and intramolecular hydrogen bonds in (A) CHARMM and (B) Drude simulations, shown as an average over three replicates. The lighter shading indicates the standard deviation across the three replicates. Hydrogen bonds formed between each amylose chain and water are shown for (C) CHARMM and (D) Drude simulations.

## Conclusions

In this study, we investigated the structural, hydration, and electronic properties of amylose and cellulose through MD simulations using CHARMM and Drude FFs. Our findings highlight the importance of the FF selection in modeling the conformational dynamics, hydration behavior, and electrostatic properties of the amylose chains in aqueous solution. Structural analyses revealed that amylose is more flexible than cellulose, and that the Drude FF samples a larger conformational space, likely due to its directional electrostatics (modeled via induced dipoles) and lower intrinsic energy barriers. End-to-end distance, radius of gyration, glycosidic torsion angles, and ring puckering corroborated that amylose adopts a more flexible conformation using the Drude FF, but cellulose adopts a relatively rigid conformation with both FFs.

Hydration analysis revealed distinct differences in water interactions between the two FFs. The CHARMM FF produced more pronounced hydration of hydroxyl groups and stronger hydrogen bonding networks with water molecules, whereas the Drude FF preferred more intramolecular hydrogen bonding in amylose. These distinctions emphasize the importance of polarization effects in governing solvation and hydrogen bond dynamics in carbohydrate systems. Additional theoretical and experimental work should be conducted to test these predictions.

Electronic property calculations provided insight into the role played by polarization in carbohydrate-water interactions. The Drude model captured variable dipole moments, giving rise to a more dynamic electrostatic environment. Comparing dipole moments of water confined inside the amylose helix to water present in the first hydration shell indicated stronger directionality with CHARMM, but with Drude, water molecules were more dynamic and heterogeneous in their orientation. Calculation of the electric field along the amylose helical axis also revealed periodic variation in electrostatic forces.

Our extension of simulations of double-helical amylose structures confirmed the stability of the helical structure, with some FF-dependent variations in transient disorder. CHARMM simulations produced more interchain hydrogen bonding, therefore favoring structural stability, whereas Drude simulations favored increased intrachain hydrogen bonding, suggesting greater flexibility. The structural stability of the double helix was generally preserved for both FFs, consistent with experimental findings.

Overall, this comparative FF study provides a systematic, molecular-level understanding of amylose and cellulose in water. The results emphasize the significance of selecting an appropriate FF based on the target properties in carbohydrate research. Future studies using enhanced sampling techniques may provide additional insights into these complex biomolecules and potentially enable improved FF parametrization and use in biomaterials engineering, drug delivery, and carbohydrate-based therapeutics.

## Supplementary Material



## Notes

RMSD of unrestrained amylose replicates; Davies–Bouldin index vs cluster number for amylose and cellulose; frame distribution among MDANCE clusters for amylose; representative cluster centroids for amylose via NANI; RMSD of cellulose replicate simulations; representative cluster centroids for cellulose; *R*_ee_ and *R*_g_ for both amylose and cellulose; water RDF and hydrogen bonding analyses per functional group in both single-chain and double-helical amylose (PDF)
