# Supplementary material for: Insights into the Electronic and Structural Properties of Cellulose and Amylose: A Comparative Force Field Study
Source: J Phys Chem B. 2026 Jan 6;130(8):2343–60. doi: 10.1021/acs.jpcb.5c07277 (PMC12951564; doi:10.1021/acs.jpcb.5c07277)
Supplement: Supplementary file 1 [file jp5c07277_si_001.pdf]

SUPPORTING INFORMATION

Insights into the Electronic and Structural Properties of Cellulose and Amylose: A Comparative Force Field Study

Esmat Mohammadi[1,2] and Justin A. Lemkul[2,3,*]

[1] Department of Chemical Engineering
[2] Department of Biochemistry
[3] Center for Drug Discovery

Virginia Tech, Blacksburg VA 24061, United States of America

SUPPORTING FIGURES

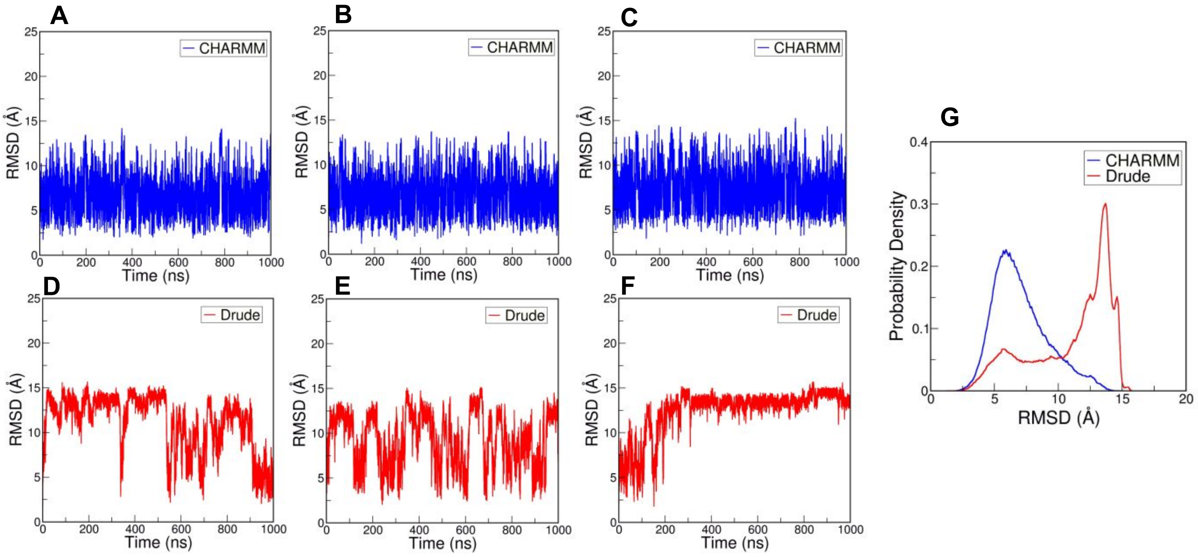

Figure S1. The root-mean-squared deviation (RMSD) of the three replicate simulations of unrestrained amylose is presented. Panels A-C correspond to simulations performed using CHARMM force fields, while panels D-F depict the RMSD of the three replicate simulations using the Drude force field. The ensemble RMSD probability densities are shown in panel G.

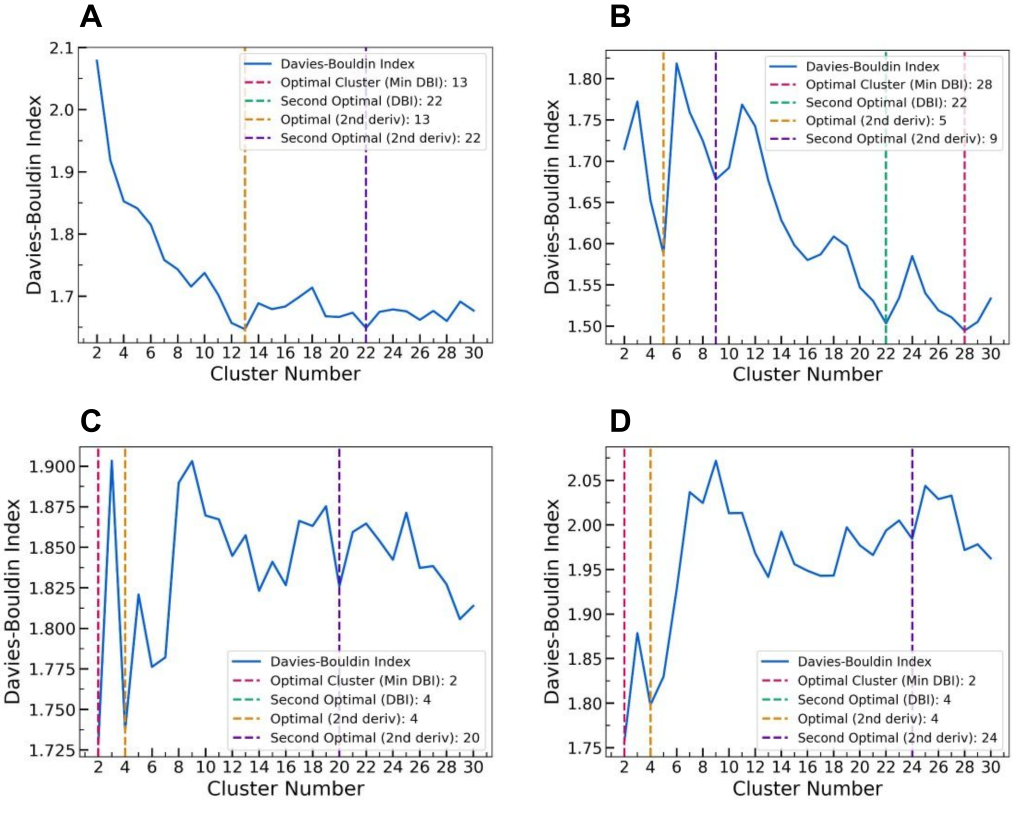

Figure S2. Davies-Bouldin (DB) index values plotted against the number of clusters used in the MDANCE analysis for (A) amylose–CHARMM, (B) amylose–Drude, (C) cellulose–CHARMM, and (D) cellulose–Drude simulations

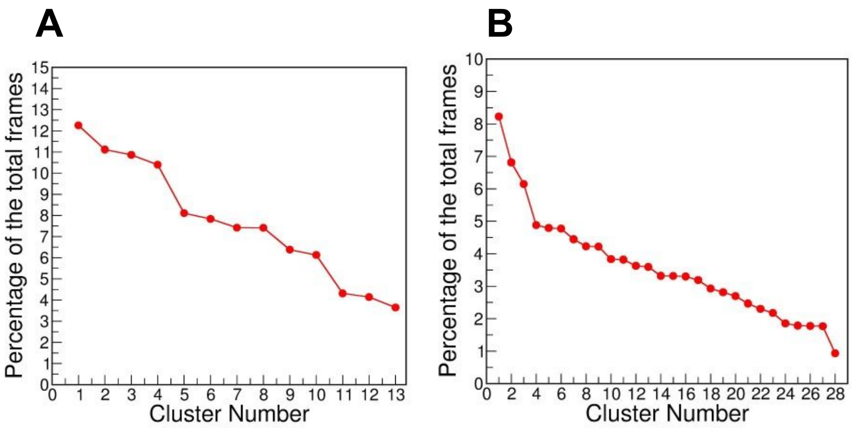

Figure S3. Distribution of frames among clusters identified by MDANCE for the amylose simulations using (A) CHARMM and (B) Drude force fields. Each bar represents the percentage of total frames assigned to a given cluster

Cluster number	1	2	3	4
Representative structure	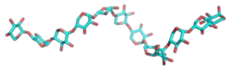	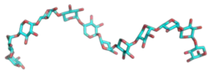	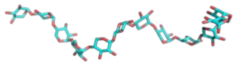	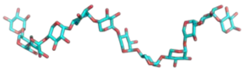
Cluster number	5	6	7	8
Representative structure	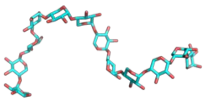	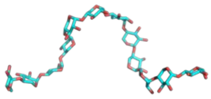	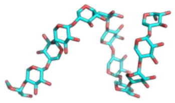	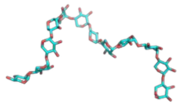
Cluster number	9	10	11	12
Representative structure	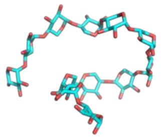	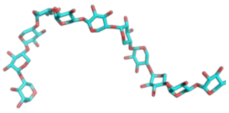	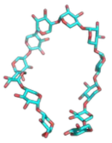	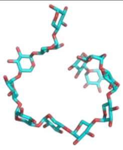
Cluster number	13			
Representative structure	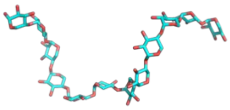			

Figure S4. Representative structures (centroids) of all clusters obtained using the NANI method for amylose simulated with the CHARMM force field.

Cluster number	1	2	3	4
Representative structure	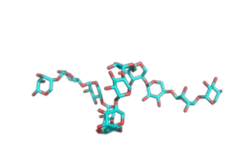	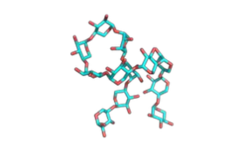	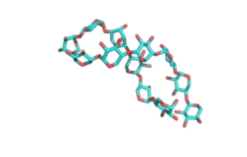	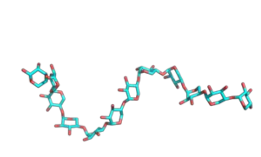
Cluster number	5	6	7	8
Representative structure	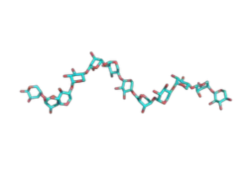	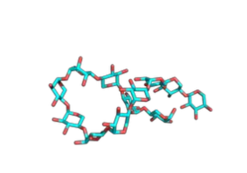	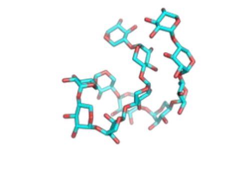	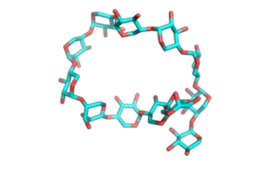
Cluster number	9	10	11	12
Representative structure	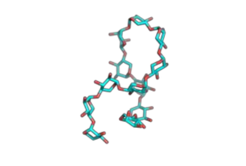	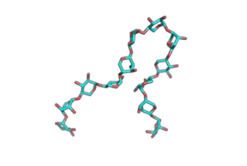	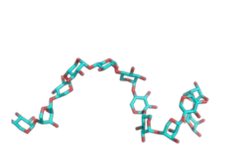	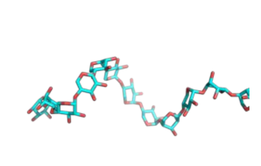
Cluster number	13	14	15	16
Representative structure	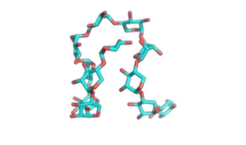	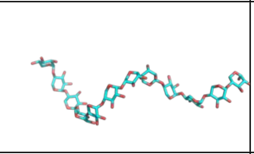	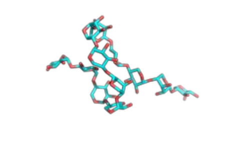	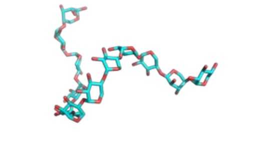
Cluster number	17	18	19	20
Representative structure	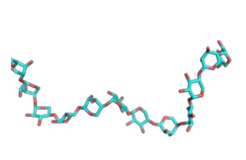	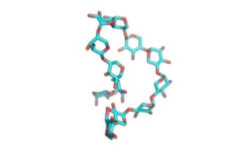	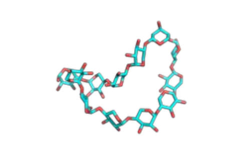	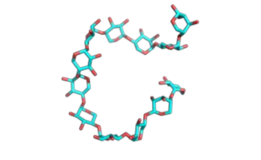
Cluster number	21	22	23	24
Representative structure	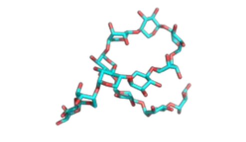	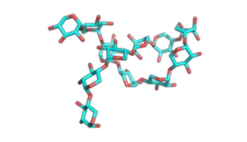	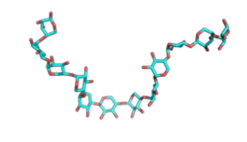	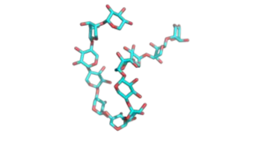
Cluster number	25	26	27	28
Representative structure	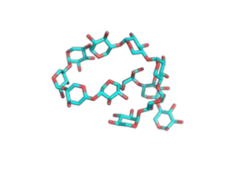	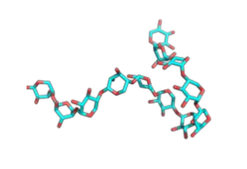	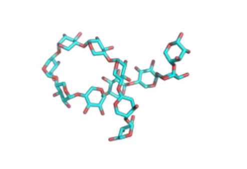	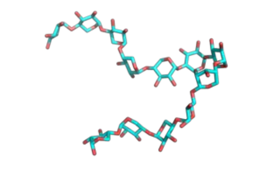

Figure S5. Representative structures (centroids) of all clusters obtained using the NANI method for amylose simulated with the Drude force field.

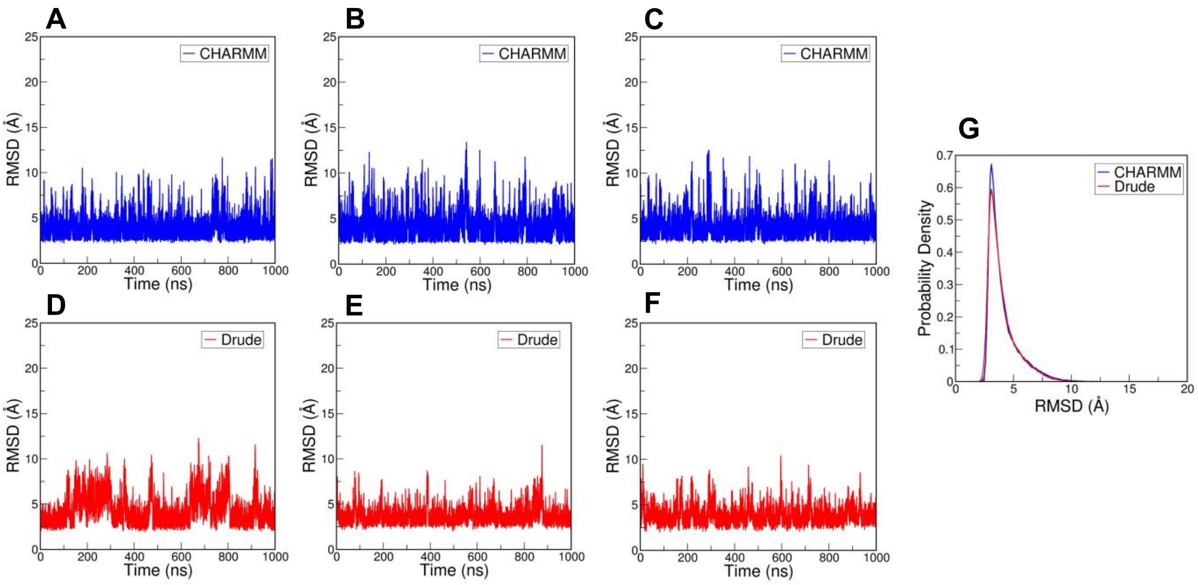

Figure S6. RMSD of the three replicate simulations of cellulose is shown. Panels A-C correspond to simulations performed using CHARMM force fields, while panels D-F illustrate the RMSD of the three replicate simulations using the Drude force field. The ensemble RMSD probability densities are shown in panel G.

Cluster number	1	2
Representative structure	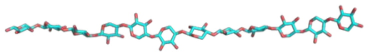	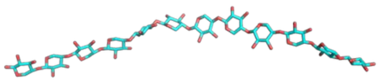

Figure S7. Representative structures (centroids) of all clusters obtained using the NANI method for cellulose chain simulated with the CHARMM force field.

Cluster number	1	2
Representative structure	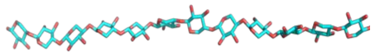	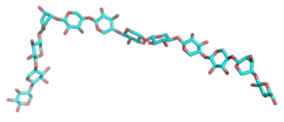

Figure S8. Representative structures (centroids) of all clusters obtained using the NANI method for cellulose chain simulated with the Drude force field.

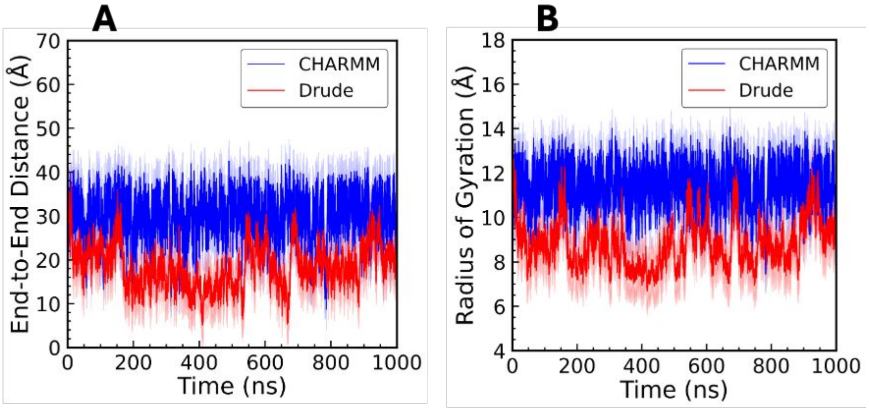

Figure S9. Structural characterization of CHARMM and Drude simulations of the unrestrained amylose chain. (A) Average end-to-end distance and (B) average radius of gyration over simulation time, calculated across three replicates. The lighter red and blue shades represent the standard deviation at each time step.

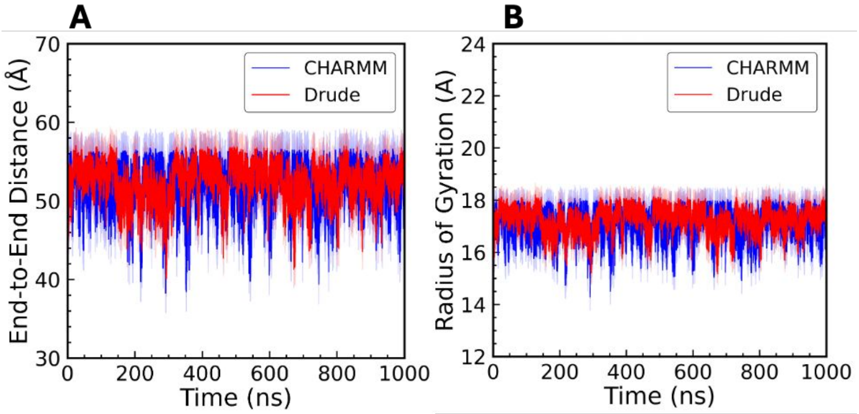

Figure S10. Structural characterization of CHARMM and Drude simulations of the cellulose chain. (A) Average end-to-end distance and (B) average radius of gyration over simulation time, calculated across three replicates. The lighter red and blue shades represent the standard deviation at each time step.

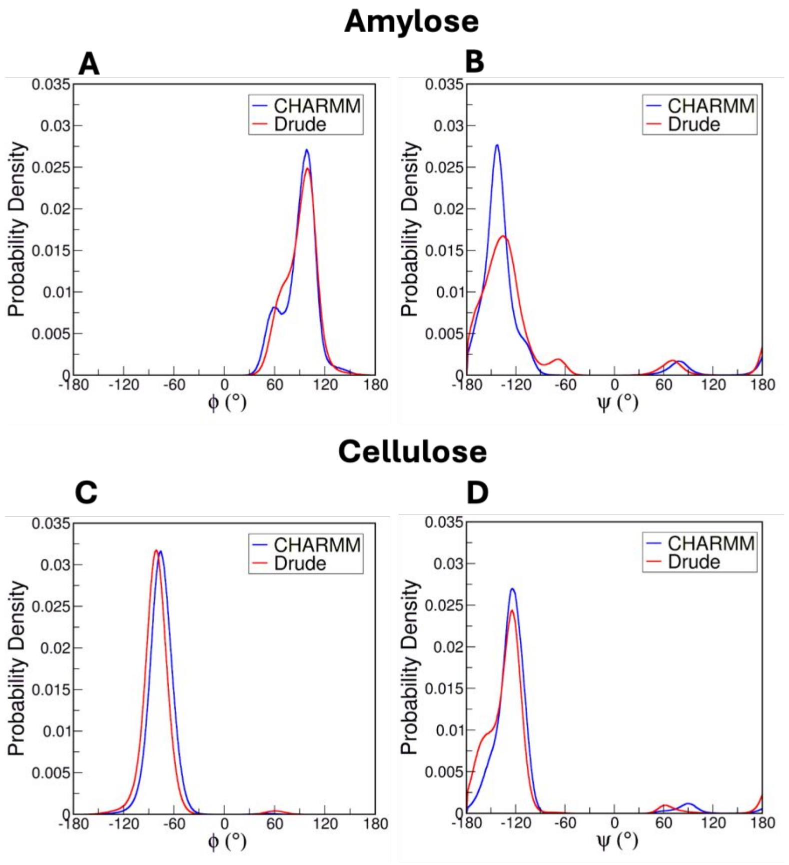

Figure S11. Characterization of glycosidic torsion angles in amylose and cellulose. Probability densities of (A) Φ and (B) Ψ for unrestrained amylose, (D) Φ and (E) Ψ in cellulose.

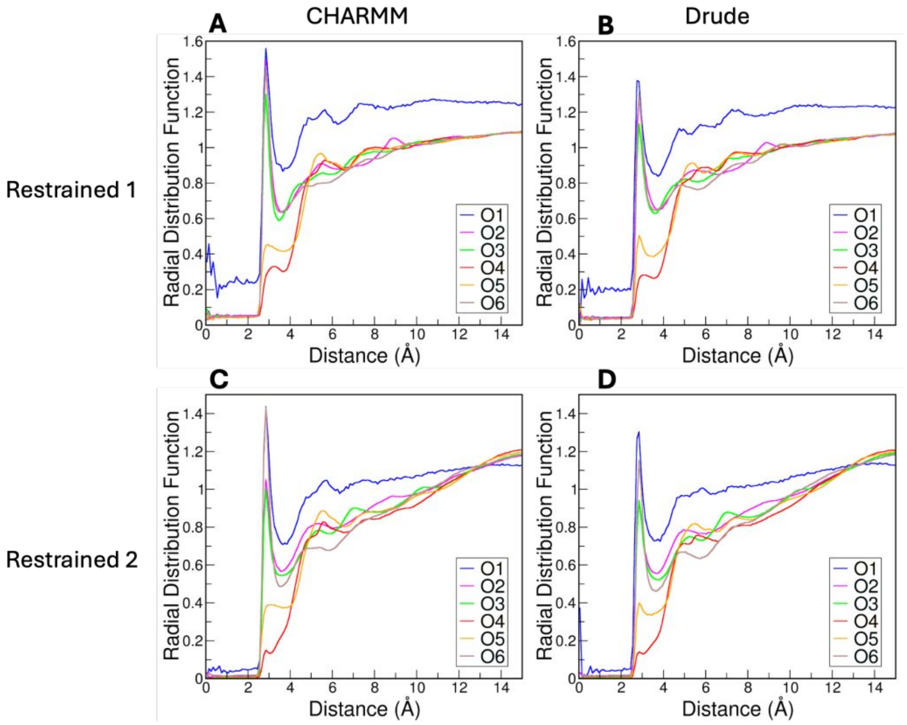

Figure S12. Radial distribution functions for water around each oxygen atom in the restrained amylose simulations. "Restrained 1" simulations with (A) CHARMM and (B) Drude FFs. "Restrained 2" simulations with (C) CHARMM and (D) Drude FFs.

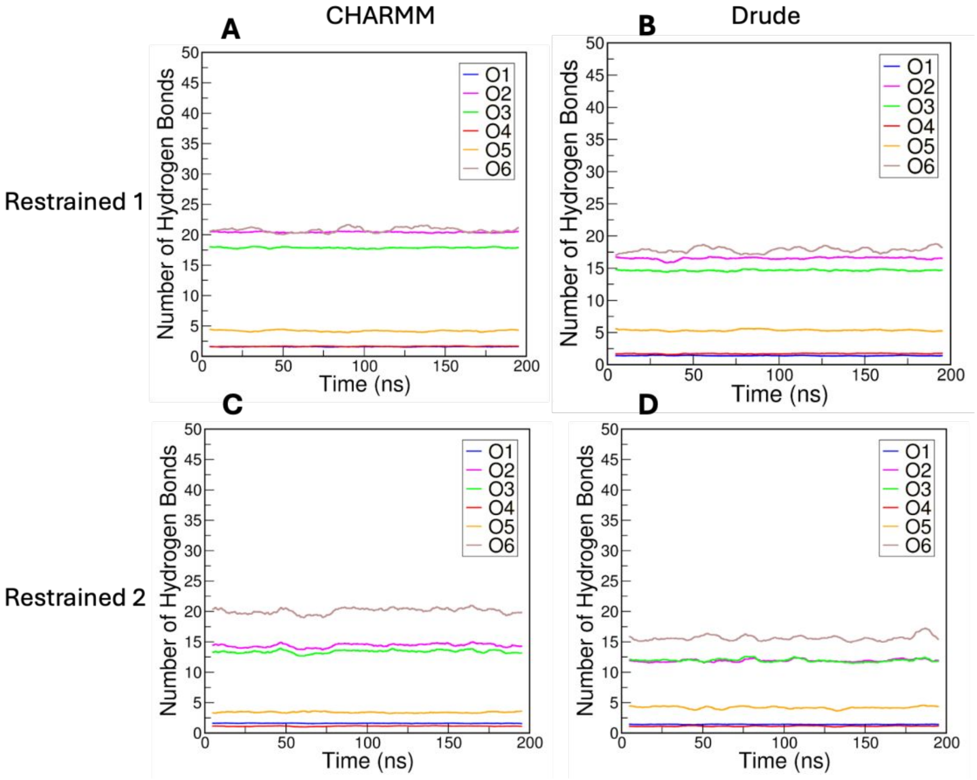

Figure S13. Hydrogen bonding in restrained, single-chain amylose simulations. Hydrogen bonds formed by each hydroxyl group and water for (A) CHARMM and (B) Drude FFs in the "restrained 1" simulations, and (C) CHARMM and (D) Drude FFs for the "restrained 2" simulations.

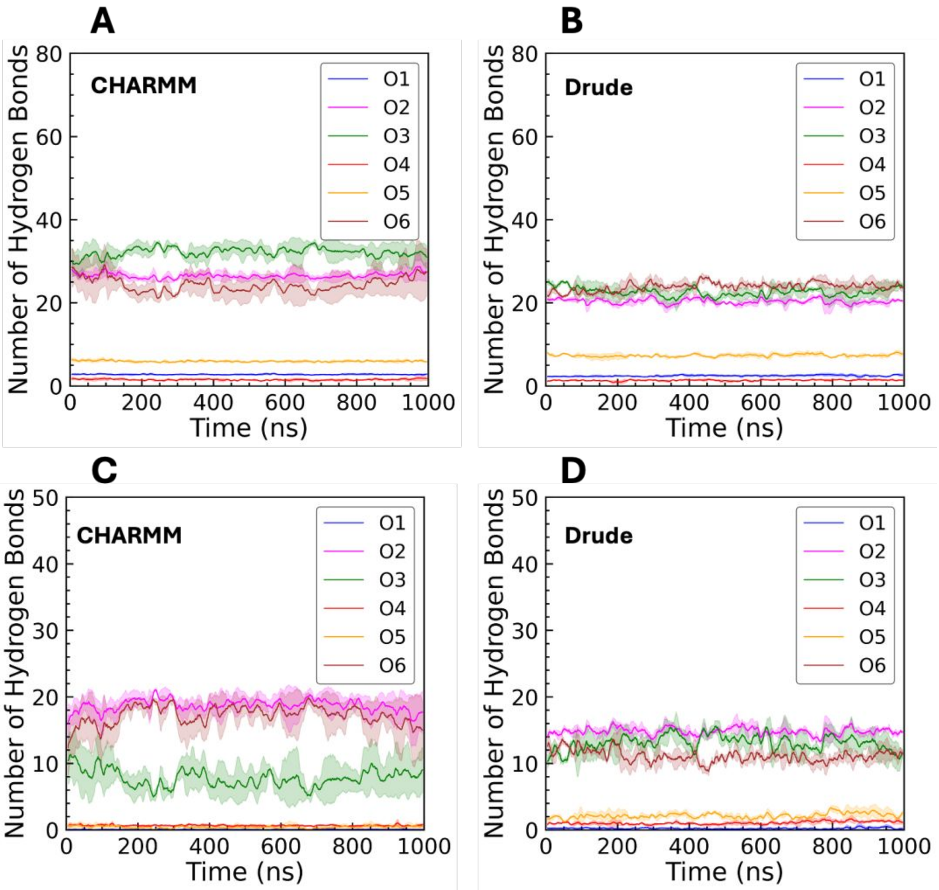

Figure S14. Hydrogen bonding in the double-helical amylose system. Hydrogen bonds formed between each hydroxyl group and water for (A) CHARMM and (B) Drude FFs. Intramolecular hydrogen bonding for each hydroxyl group in (C) CHARMM and (D) Drude FFs. All plots show the average number of hydrogen bonds across three replicates, with lighter colors indicating the standard deviation across the replicates.
